# Interpretable Deep Learning of Molecule‐Solvent Interactions Using a Solvatochromic Subgroup Contribution Framework

**DOI:** 10.1002/advs.77347

**Published:** 2026-08-27

**Authors:** Jinyong Park, Minhi Han, Kiwoong Lee, Seokwoo Kim, Sungnam Park

**Affiliations:** ^1^ Department of Chemistry Korea University Seoul South Korea; ^2^ Deep4Chem Inc. Seongbuk‐gu Seoul South Korea

**Keywords:** electronic transition, group contribution, hierarchical molecular graph neural networks, interpretable deep learning, solvatochromism

## Abstract

Solvent effects critically influence the photophysical and optical properties of molecules in solution, yet a quantitative and interpretable description of solvent effects remains a long‐standing challenge. In this study, to quantitatively understand and chemically interpret solvatochromism, deep learning (DL) models based on the solvatochromic subgroup contribution (SSC) framework were introduced, which describes solvatochromic shifts through localized interactions between molecular subgroups and the surrounding solvent. In this framework, the electronic transition energies of molecules in solution are successfully modeled based on three chemically interpretable contributions: the reference property (RP), subgroup contribution (SC), and proximity effect factor (PEF), providing a chemistry‐informed representation of solvent‐dependent electronic transition energies. Trained on an experimental database of solvent‐dependent absorption and emission energies, SSC‐based deep learning models achieve both high prediction accuracy and subgroup‐level interpretability of solvatochromic shifts. More importantly, the learned SC values show strong correlations with classical solvent polarity parameters (i.e., *E*_T_(30) and Kamlet‐Taft parameters) and reproduce Bilot‐Kawski relationships, confirming that the SSC framework captures explicit and physically meaningful molecule‐solvent interactions. The SSC framework is a scalable and generalizable tool for modeling molecule‐solvent interactions in chemistry and materials science, providing improved predictive accuracy and chemical interpretability.

## Graphical abstract

Chemistry‐informed deep learning (DL) models based on the solvatochromic subgroup contribution (SSC) framework predict and interpret solvent‐dependent spectral shifts, revealing physically meaningful subgroup‐solvent interactions.

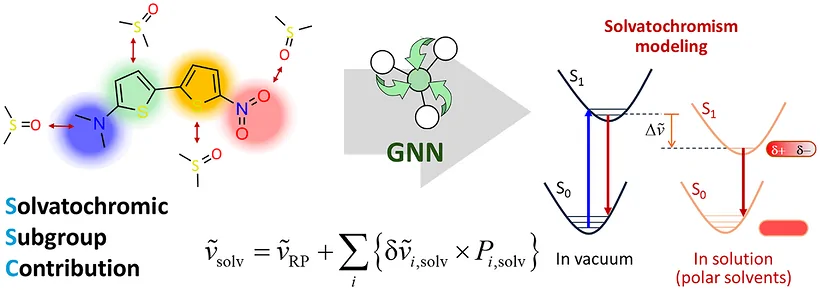

## 1 Introduction

Solvatochromism [1, 2], the phenomenon by which the optical properties of molecules vary with the surrounding solvent, is of broad interest across disciplines including chemistry, materials science, pharmaceuticals, and environmental science. Although solvatochromic data can be readily obtained through accessible spectroscopic methods such as UV–vis absorption and photoluminescence spectroscopy, such measurements yield profound insights into the intermolecular interactions between molecules and the surrounding solvent [3]. These insights not only deepen our understanding of solvent effects on molecular energetics, but also help elucidate reaction mechanisms in solution and interpret solvent‐induced modulations of the electronic states of molecules in solution.

Over the past decades, numerous studies have sought to unravel the mechanisms underlying solvatochromism [3, 4, 5, 6, 7, 8, 9, 10]. It is common for organic chemists to report solvatochromic responses of newly synthesized molecules to understand their optical properties. In most cases, solvatochromism has been interpreted using empirical solvent polarity parameters, most notably Reichardt's *E*_T_(30) [5, 6, 10] and Kamlet‐Taft parameters [7, 8, 9] (*α*, *β*, and π*), which quantify solvent effects through the spectral behavior of reference dyes. Although these solvent polarity parameters have been invaluable for generalizing solvent effects on optical properties, they inevitably rely on simplified representations of complex molecule‐solvent interactions [2]. Solvatochromic shifts are governed by multiple factors, including solvent polarity, hydrogen‐bonding ability [11], acidity/basicity [9], and viscosity [12]. As the empirical parameters are typically derived from the optical responses of specific indicator dyes (i.e., Betaine‐30 [6]), their applicability may be limited in capturing the full complexity of molecule‐solvent interactions across structurally diverse molecular systems.

To account for solvent effects in first‐principles DFT calculations, continuum solvation models, such as the polarizable continuum model (PCM) [13] and solvation model based on density (SMD) [14], have been widely employed. While these models efficiently incorporate solvent effects by treating the solvent as a homogeneous, polarizable dielectric medium, they inherently neglect explicit solute–solvent interactions. Although such specific interactions can be captured by explicitly including solvent molecules via approaches like QM/MM, the associated computational cost severely limits their practical application.

Recently, artificial intelligence (AI) has been rapidly applied to many research fields, accelerating research and facilitating data‐driven discovery [15, 16, 17, 18, 19, 20]. Among various AI approaches, graph neural networks (GNNs) have proven to be particularly effective for predicting a wide range of molecular properties. When developing models to predict solvent‐dependent molecular properties, a central challenge lies in effectively representing molecule‐solvent interactions within an AI framework. The most common and straightforward strategy involves concatenating the learned representations (or context vectors) of the molecule and the solvent. In our recent studies, we also adopted this approach to predict optical and photophysical properties [21, 22, 23] as well as electrochemical properties [24]. However, this concatenation strategy treats the molecule and solvent as largely independent entities, neglecting the explicit interactions that govern their coupled behavior. To overcome this limitation, several interaction‐aware models have been introduced. Pathak et al. proposed a chemically interpretable graph interaction network (CIGIN), where the interaction map framework was introduced to combine the molecule and solvent features via dot product to account for the molecule‐solvent interactions [25]. CIGIN achieved improved accuracy in the prediction of solvation free energy compared to concatenation‐based approaches. Ramani et al. proposed MolMerger, a framework that merges molecule and solvent graphs into a single molecular entity, based on the assumption that molecule‐solvent interactions are governed by atomic partial charges, enabling improved prediction of solubility [26]. While these approaches move toward more explicit representations of molecule‐solvent interactions, their ability to yield chemically interpretable insights into the origins of solvent effects remains limited. Ideally, a promising strategy to improve both model performance and interpretability is to design a chemistry‐informed architecture that intrinsically reflects chemical knowledge into its computational process [19].

In this study, we introduce the solvatochromic subgroup contribution (SSC), which describes solvatochromic shifts through localized interactions between molecular subgroups and the surrounding solvent. In conventional concatenation‐based approaches, solvatochromic shifts of molecules in solution are estimated from the overall molecule‐solvent interaction [27, 28, 29]. In contrast, the SSC framework assumes that solvatochromic shifts arise from the cumulative contributions of individual molecular subgroups interacting with their solvent environments. Accordingly, the SSC framework models the electronic transition energies of molecules in solution as the sum of three chemically interpretable contributions: the reference property (RP), subgroup contribution (SC), and proximity effect factor (PEF), providing a chemistry‐informed representation of solvent‐dependent electronic transition energies. To validate this concept, we developed deep learning (DL) models based on the SSC framework and trained them on an extensive experimental database of solvent‐dependent absorption and emission energies. The SSC‐based DL models demonstrate substantial improvements in both predictive accuracy and chemical interpretability compared with existing approaches. Furthermore, using the best‐performing SSC‐based model, we introduce the normalized subgroup contribution (NSC) as a quantitative descriptor for subgroup‐level solvent responses, offering a chemically interpretable metric for understanding how subgroups (or functional groups) contribute to solvent‐dependent changes in electronic transition energies. Comparative analyses with traditional empirical solvent polarity parameters confirm that the SSC framework captures explicit and physically meaningful molecule‐solvent interactions. Overall, by embedding physical insight into deep learning architectures, the SSC framework makes it possible to realize explainable and chemistry‐informed AI models, enabling mechanistic understanding of solvatochromic phenomena.

## 2 Results

### 2.1 Implementation of the SSC‐Based Model

**FIGURE 1:**
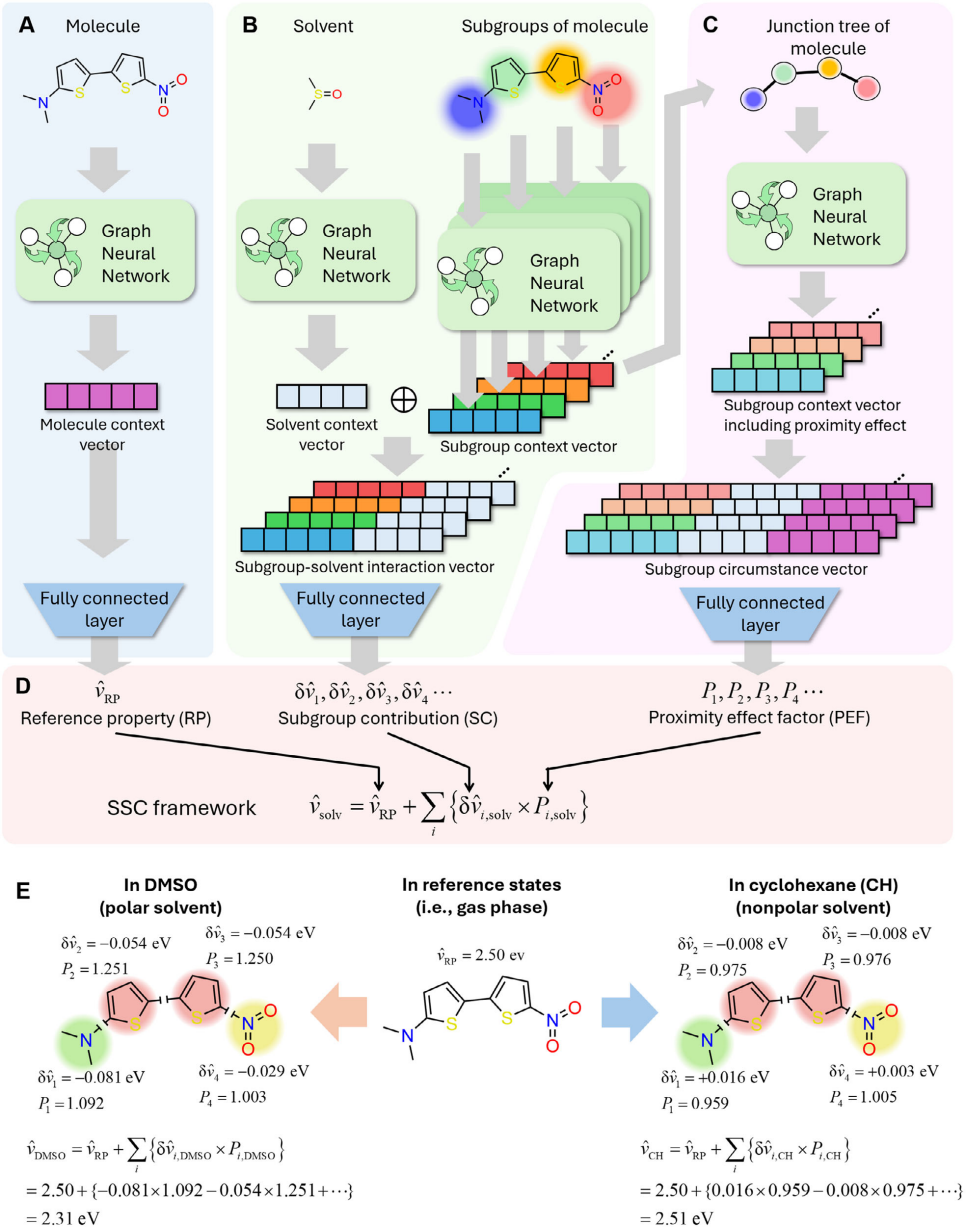
Overall architecture of SSC‐based DL model. The model incorporates three distinct contributions: (A) Reference property (RP) representing the intrinsic electronic transition energy in the gas‐phase, (B) Subgroup contribution (SC) capturing localized subgroup‐solvent interactions, and (C) Proximity effect factor (PEF) accounting for the influence of neighboring subgroups. (D) Formal expression of the SSC framework for calculating solvent‐dependent electronic transition energies. (E) Representative example of SSC‐based prediction of emission energies of a molecule in *N*,*N*‐dimethyl sulfoxide (DMSO, polar) and cyclohexane (CH, nonpolar).

The overall architecture of the SSC‐based DL model is illustrated in Figure 1 (see the Supporting Information (SI) for details). Following the SSC framework, the optical properties of a molecule in a given solvent (e.g., absorption and emission energies) are expressed as the sum of three contributions. The RP (Figure 1a) is derived solely from the molecular structure and represents an intrinsic molecular property independent of solvent effects. The SC (Figure 1b) arises from interactions between individual subgroups and the solvent. Notably, SC values are calculated independently of the overall molecular structure, ensuring that a specific subgroup yields a consistent contribution in a given solvent. The PEF (Figure 1c) accounts for modifications of subgroup contributions due to the influence of adjacent subgroups, such as inductive effects, resonance delocalization, or steric hindrance.

### 2.2 Segmentation of Molecules into Subgroups

Molecular segmentation was performed using the algorithm introduced in our previous work [30]. A key advantage of this algorithm is its flexibility, as the segmentation resolution can be controlled using three integer parameters (*s*,*c*,*a*). In this study, we employed the segmentation rule (6,2,0), which generates subgroups that closely correspond to conventional organic functional groups, as illustrated in Figure S12. This choice is supported by the accurate and stable predictive performance achieved with this scheme, as summarized in Table S10. Moreover, the chemically intuitive nature of the subgroups generated using the segmentation rule (6,2,0) facilitates straightforward interpretation of the corresponding subgroup contributions.

### 2.3 Perspective of the SSC Framework on Solvatochromism

The primary strength of the SSC framework lies in its interpretability, as it enables solvent effects on molecular properties to be quantified at the level of individual subgroups. In principle, the electronic transition energies of a molecule are intrinsic properties that can be directly determined by electronic transition spectroscopy, such as UV–vis absorption and photoluminescence measurements. In this study, we approximate the reference property (RP, ${\tilde{\nu }}_{\mathrm{RP}}$) in Figure 1 as the gas‐phase value (${\tilde{\nu }}_{\mathrm{gas}}$)

(1) $${\tilde{\nu }}_{\mathrm{RP}}={\tilde{\nu }}_{\mathrm{gas}}$$

Although the RP corresponds to the gas‐phase value in principle, within the SSC framework it is regarded as a latent intrinsic property inferred by DL models. For most organic molecules, however, electronic transition energies are typically measured in solution, where molecule‐solvent interactions (or solvation effects) induce red or blueshifts due to stabilization or destabilization of electronic states. This solvent‐induced deviation from the gas‐phase value (${\tilde{\nu }}_{\mathrm{gas}}$) is referred to as the solvatochromic shift ($\Delta {\tilde{\nu }}_{\mathrm{solv}}$). Accordingly, the electronic transition energy of a solvated molecule (${\tilde{\nu }}_{\mathrm{solv}}$) can be expressed as

(2) $${\tilde{\nu }}_{\mathrm{solv}}={\tilde{\nu }}_{\mathrm{RP}}+\Delta {\tilde{\nu }}_{\mathrm{solv}}$$

In this study, we further assume that $\Delta {\tilde{\nu }}_{\mathrm{solv}}$ can be approximated as the sum of the contributions from individual subgroup‐solvent interactions, hereafter termed subgroup contribution (SC, $\delta {\tilde{\nu }}_{i,\mathrm{solv}}$). To avoid ambiguity, we distinguish the solvatochromic shift of the entire molecule ($\Delta {\tilde{\nu }}_{\mathrm{solv}}$) and the individual subgroup contribution ($\delta {\tilde{\nu }}_{i,\mathrm{solv}}$). Furthermore, we recognize that subgroup contributions are not always independent but can be modulated by the presence of adjacent subgroups. To capture this, we introduce a proximity effect factor (PEF), which accounts for perturbations in subgroup contributions arising from electronic inductive effects by electron‐withdrawing or electron‐donating groups, resonance delocalization by extended π‐conjugation, and steric hindrance from bulky groups. Under this assumption, the solvatochromic shift of a molecule in a given solvent can be expressed as,

(3) $$\Delta {\tilde{\nu }}_{\mathrm{solv}}=\sum_{i}\left\{\delta {\tilde{\nu }}_{i,\mathrm{solv}}\times P_{i}\right\}$$

where $\delta {\tilde{\nu }}_{i,\mathrm{solv}}$ and *P_i_* represent the subgroup contribution (SC) and the proximity effect factor (PEF) of the *i*th subgroup, respectively. The SSC‐based DL model (Figure 1) was designed to estimate RP (${\tilde{\nu }}_{\mathrm{RP}}$), SC ($\delta {\tilde{\nu }}_{i,\mathrm{solv}}$), and PEF (*P_i_*) values for absorption and emission energies of molecules in solvents. As illustrated in Figure 1e, the DL model integrates these contributions to reconstruct the experimentally observed electronic transition energies of molecules in different solvents. Throughout this work, a circumflex (ˆ) denotes a model‐predicted quantity. Accordingly, the predicted RP, SC, and PEF values are denoted by ${\hat{\nu }}_{\mathrm{RP}}$, $\delta {\hat{\nu }}_{i,\mathrm{solv}}$, and ${\hat{P}}_{i}$, respectively, to distinguish them from their corresponding ideal values.

### 2.4 Predictive Performance of DL Models

**FIGURE 2:**
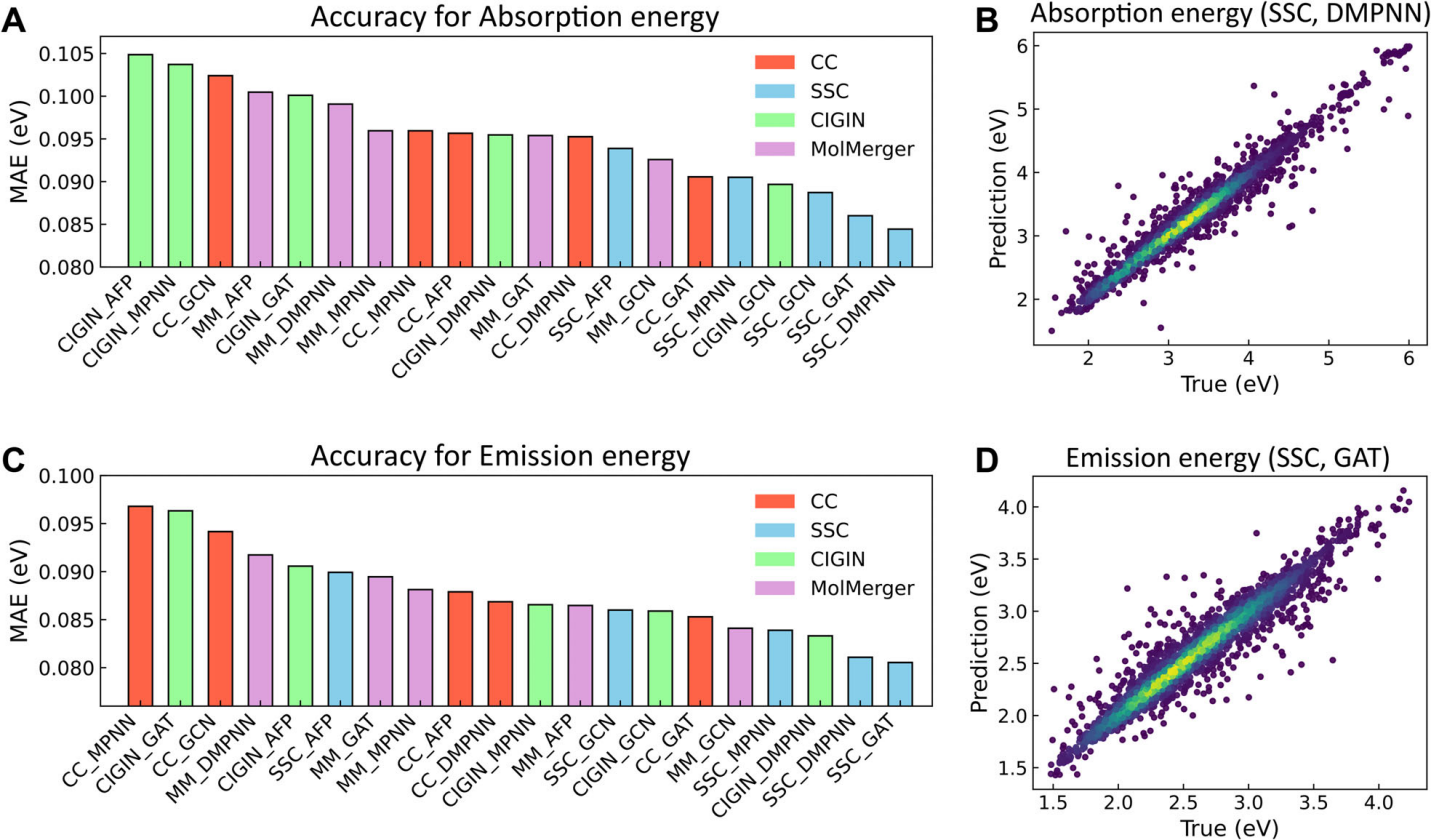
Predictive performance of SSC‐based models compared with benchmark models. (A) Mean absolute error (MAE) of absorption energy predictions for the concatenation (CC), SSC, CIGIN, and MolMerger (MM) methods. (B) Parity plot of predicted vs. experimental absorption energies using the SSC‐DMPNN model. (C) MAE of emission energy predictions for the same set of models. (D) Parity plot of predicted vs. experimental emission energies using the SSC‐GAT model. Exact metric values are provided in Table S1.

In this study, we directly benchmarked the SSC framework against the concatenation (CC) method as well as two other recently proposed architectures: CIGIN and MolMerger. For graph neural networks, we benchmarked 5 well‐known algorithms for each solvent‐incorporating method: GCN [31], MPNN [32], DMPNN [33], GAT [34], and AttentiveFP [35]. Figure 2 summarizes the predictive performance of these models trained on our curated database of absorption and emission energies. As shown in Figure 2, SSC‐based models consistently outperformed other approaches. In particular, the SSC‐DMPNN network achieved the best predictive accuracy for absorption energies, with a mean absolute error (MAE) of 0.0844 eV, while the SSC‐GAT network achieved the best accuracy for emission energies, with an MAE of 0.0805 eV. The performance of the CIGIN method was highly variable depending on the choice of underlying graph neural network, ranging from the second‐best to the worst model. The MolMerger approach showed modest improvements over the CC method but remained inferior to the SSC framework. Taken together, these results demonstrate that explicitly modeling solvent effects as subgroup‐solvent interactions within the SSC framework yields superior predictive performance compared to benchmarking models, suggesting that the SSC framework provides a reliable basis for incorporating solvent effects in solvatochromism.

Additionally, Table 1 shows the SSC‐GAT model predictions for a series of recently reported chromophores, including derivatives of pyrene [36], pyrrolopyrrole [37], cyanostilbene [38], BODIPY [39], and squaraine [40]. Importantly, none of these molecules were included in the training, validation, or test datasets. The transition energies of these chromophores cover a range from 360 to 630 nm for absorptions and from 407 to 580 nm for emissions. Despite the diversity, our model accurately reproduces the absolute transition energies and the solvatochromic trends across a variety of solvents.

**TABLE 1:** Prediction results of the SSC‐GAT model for the recently reported chromophores.

Molecule	Solvent	Exp. *λ*_abs_ (nm)	Pred. *λ*_abs_ (nm) [Δ (nm)]^a^	Exp. *λ*_emi_ (nm)	Pred. *λ*_emi_ (nm) [Δ (nm)]^a^	Refs.
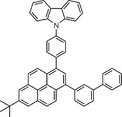	ACN	360	365 [5]	421	424 [3]	[32]
	DMF	363	366 [3]	426	426 [0]	
	DMSO	367	366 [1]	431	429 [2]	

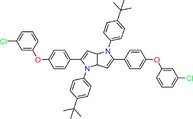	Tol	357	361 [4]	435	423 [12]	[33]
	THF	358	358 [0]	437	433 [4]	
	DMSO	353	359 [6]	442	442 [0]	

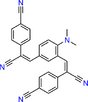	EA	390	392 [2]	556	546 [10]	[34]
	DMF	403	398 [5]	580	569 [11]	
	EtOH	391	394 [3]	567	565 [2]	

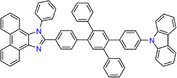	Tol	362	356 [4]	407	397 [10]	[35]
	Clf	—	352	415	419 [4]	
	DMF	—	354	432	433 [1]	

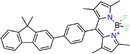	Hx	500	502 [2]	518	522 [4]	[36]
	DCM	500	504 [4]	520	536 [16]	
	MeOH	497	504 [7]	516	527 [11]	

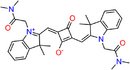	Clf	633	624 [9]	—	687	[37]
	DCM	633	634 [1]	—	688	

Abbreviations: ACN: acetonitrile; DMF: *N*,*N*‐dimethylformamide; DMSO: dimethylsulfoxide: Tol: toluene; THF: tetrahydrofuran; EA: ethyl acetate; EtOH: ethanol; MeOH: methanol; Clf: chloroform; Hx: hexane; DCM: dichloromethane.

^a^ Δ **=** Predicted value − Experimental value. The average absolute Δ values for the absorption (*λ*_abs_) and emission (*λ*_emi_) wavelengths are 3.4 and 6.0 nm, respectively.

### 2.5 Ablation Study of the SSC Framework

An ablation study was performed using the SSC‐GAT model to assess the significance of the PEF term, with results summarized in Table S3. When the PEF was excluded (denoted SSC/PE), prediction accuracy dropped significantly compared to the full SSC model, confirming the necessity of accounting for proximity effects. However, a potential concern is that SC values may be inherently influenced by PEF contributions, which could reduce the rigidity and thus the interpretability of the SC terms. To mitigate this issue, we implemented a two‐step training strategy designed to disentangle the two terms while preserving predictive performance. In the first stage, the model was trained with all PEF values fixed at 1.0, thereby isolating and optimizing subgroup contributions. In the second stage, the SC layers were frozen, and the PEF values were subsequently optimized (denoted Frozen‐SSC in Table S3). This approach preserved prediction accuracy at a level comparable to the full SSC model while enhancing the reproducibility and chemical interpretability of the SC values.

### 2.6 Chemical Validity Evaluation of the SSC Framework

The SSC framework assumes that each subgroup independently interacts with the solvent and that their contributions additively determine the overall electronic transition energies. While the PEF is introduced as a correction factor, the RP and SC terms are expected to result from chemical contributions of the corresponding molecular subgroups. To assess the validity of this assumption, we consider three necessary conditions.

1. Additivity of subgroup contributions: The underlying assumption of SSC, which is that solvatochromic shifts can be approximated as the sum of subgroup‐solvent interactions, is chemically difficult to validate directly. Nevertheless, this assumption is at least justifiable from a DL perspective, as evidenced by the consistently improved predictive performance of SSC‐based DL models.
2. Reproducibility of derived values: For RP and SC to be chemically meaningful, the values obtained from trained models must be reproducible across independent training runs [41]. If the results were purely random, they would have no chemical or physical significance.
3. Consistency with chemical knowledge: Finally, the derived contributions should align with established empirical trends in solvatochromism, reflecting broadly observed chemical rules.

### 2.7 Evaluation of the Reproducibility of SSC Parameters

To evaluate the reproducibility of the SSC parameters, we examined the trends in the RP and SC values obtained from models initialized with different random seeds. Consistent RP and SC values across independently trained models would indicate that these parameters capture chemically meaningful information associated with intrinsic molecular properties (i.e., gas‐phase values) and solvation effects, respectively. Detailed analyses of the reproducibility of the RP and SC values are provided in the Supporting Information. Notably, the RP values (${\hat{\nu }}_{\mathrm{RP}}$) exhibited lower reproducibility than the SC values, showing less consistent trends across models and datasets. Within the SSC framework, the RP is conceptually expected to represent the gas‐phase transition energy (${\tilde{\nu }}_{\mathrm{gas}}$). In practice, however, the RP is treated as a latent intrinsic molecular property inferred by DL models from electronic transition energies of molecules measured in solution. Consequently, the learned RP values do not necessarily correspond directly to experimentally measured gas‐phase transition energies. This limitation could be addressed by incorporating experimental gas‐phase electronic transition energies into the training dataset. However, sufficiently large and chemically diverse datasets of such measurements are currently unavailable. The reliability of the RP value also depends on the robustness of the corresponding SC values. From Equations (2) and (3), the RP value can be derived as

(4) $${\hat{\nu }}_{\mathrm{RP}}={\tilde{\nu }}_{\mathrm{solv}}-\sum_{i}\left\{\delta {\hat{\nu }}_{i,\mathrm{solv}}\times {\hat{P}}_{i}\right\}\approx {\tilde{\nu }}_{\mathrm{solv}}-\sum_{i}\delta {\hat{\nu }}_{i,\mathrm{solv}}$$

where the second expression is obtained by assuming all PEF values to be 1.0. Accordingly, when a molecule contains unique or unusual subgroups whose SC values have not been learned robustly, the resulting RP value may not reliably represent an intrinsic molecular property. Instead, it may arise from an underdetermined partitioning of the observed transition energy between the RP and SC contributions. Although mathematical constraints or modifications to the model architecture could improve the numerical reproducibility of the RP values, such approaches alone would not ensure their chemical interpretability. Future studies should therefore focus on developing physically grounded strategies that simultaneously improve the reproducibility and chemical interpretability of the RP values.

In contrast, the relative changes in SC values in various solvents exhibited strong reproducibility (Figure S6) across models, supporting the notion that SC captures chemically meaningful contributions to solvent‐dependent electronic transition energies. However, the absolute SC values were less reproducible, reflecting variability in RP predictions as mentioned in the previous section. To mitigate this limitation, we introduced the concept of normalized SC (NSC, $\delta {\hat{\nu }}^{*}$), defined as the ensemble‐averaged relative change in SC in various solvents (see the Supporting Information for details). On this basis, we further define the predicted normalized solvatochromic shift ($\Delta {\hat{\nu }}^{*}$) of a molecule, which represents the relative change of electronic transition energy with solvent and is expressed without the PEF term to enhance interpretability (see the Supporting Information for details). This normalization enables the extraction of chemically meaningful trends from SSC‐derived contributions while minimizing the confounding influence of RP variability. In the following sections, we evaluate the chemical validity of NSC from multiple perspectives. Although NSC tables for both datasets of absorption and emission energies were presented, our discussion primarily emphasizes emission energies, as solvatochromic shifts are typically more pronounced in emission spectra than in absorption spectra.

### 2.8 Validation of Normalized Subgroup Contribution (NSC) Values

Table 2 summarizes the NSC values for the absorption and emission energies ($\delta {\hat{\nu }}_{\mathrm{abs}}^{*}$ and $\delta {\hat{\nu }}_{\mathrm{emi}}^{*}$) of selected subgroups, respectively, while comprehensive results for all subgroups are provided in Supporting Files 2 and 3, repsectively. For instance, the methyl group shows relatively small $\delta {\hat{\nu }}_{\mathrm{emi}}^{*}$ in all solvents, consistent with the expectation that this nonpolar subgroup interacts weakly with solvent environments. In contrast, the amine group, a highly polar subgroup, exhibits pronounced solvent‐dependent shifts in emission energy, ranging from $\delta {\hat{\nu }}_{\mathrm{emi}}^{*}$ = –41.48 meV in methanol (polar, hydrogen‐bonding) to $\delta {\hat{\nu }}_{\mathrm{emi}}^{*}$ = +41.72 meV in toluene (nonpolar). Such strong dependence is in line with the well‐known sensitivity of the amine group to solvent effects. Correlations between NSC values and conventional solvent polarity parameters (dielectric constant and *E*_T_(30)) in Figure S11 further illustrate the validity of NSC values.

**TABLE 2:** Representative examples of NSC values for selected subgroups in different solvents.

Subgroup	Counts^a^	Absorption energies, $\delta {\hat{\nu }}_{\mathrm{abs}}^{*}$ (meV)				Emission energies, $\delta {\hat{\nu }}_{\mathrm{emi}}^{*}$ (meV)			
		Tol^b^	DCM^b^	ACN^b^	MeOH^b^	Tol^b^	DCM^b^	ACN^b^	MeOH^b^
Benzene	10 791	−11.43	−2.31	−3.94	18.74	+18.03	−6.88	−16.56	−12.38
Methyl	8138	+0.05	−3.52	+1.32	+1.53	+1.39	−1.00	+0.076	−1.76
Fluoro	3596	−3.50	−0.70	+4.69	+1.43	−7.36	+0.15	+7.76	+9.42
Nitrile	1315	−10.41	−4.82	+5.75	+7.36	−1.43	−7.04	−14.77	−5.55
Amine	651	+4.37	+25.93	+0.79	−7.59	41.72	6.18	−0.69	−41.48

*Note*: NSC values were obtained by ensemble‐averaging SC values from independently trained SSC‐GAT models. For clarity, subgroups with different substitution patterns (e.g., mono‐, ortho‐, meta‐, and para‐disubstituted benzenes) were aggregated into a single benzene group. Complete NSC values for all subgroups are provided in Supporting Files 2 and 3.

^a^ Number of molecules containing the subgroup in the experimental database.

^b^ Tol: toluene; DCM: dichloromethane; ACN: acetonitrile; MeOH: methanol.

Overall, the SSC framework allows subgroup‐level quantification of solvatochromic shifts, yielding chemically interpretable insights into molecule‐solvent interactions. By linking subgroup‐specific responses to established solvent polarity parameters, NSC not only highlights the strengths and limitations of conventional solvatochromic models but also provides a path toward more detailed and accurate descriptions of solvent effects.

**FIGURE 3:**
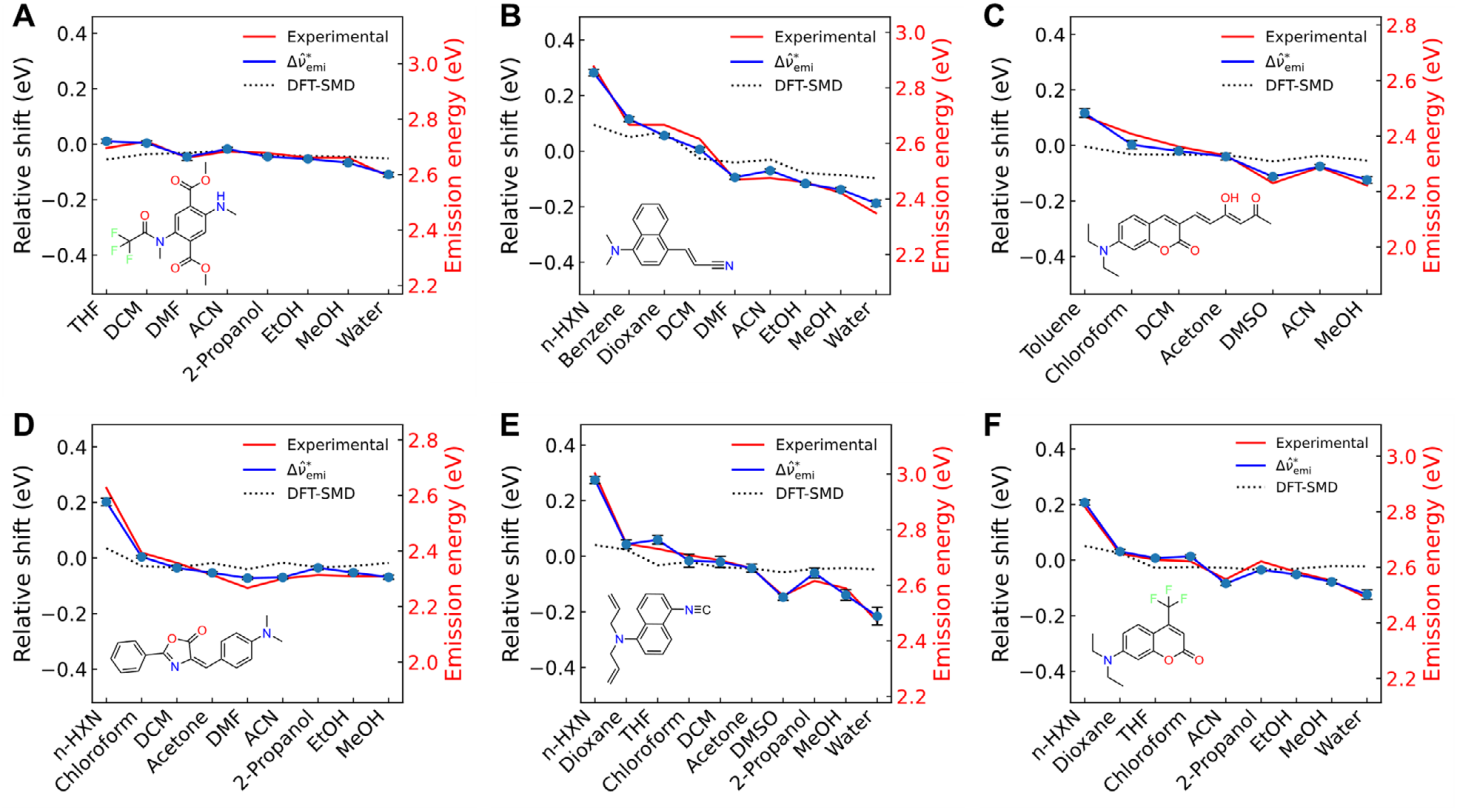
Comparison of predicted solvatochromic shifts with experimental and DFT‐calculated values [42, 43, 44, 45, 46, 47]. Shown are experimental emission energies (red solid lines), predicted normalized solvatochromic shift ($\Delta {\hat{\nu }}_{\mathrm{emi}}^{*}$, blue solid lines), and TD‐DFT‐calculated emission energy shifts (black dashed lines). For direct comparison of the solvatochromic shifts, only relative shifts are plotted, whereas the corresponding absolute experimental emission energies are presented on the right‐hand axis. The solvents on the *x*‐axis are arranged in order of polarity according to the *E*_T_(30) scale. The TD‐DFT calculations were performed at the *ω*B97XD/def2‐SVP level using S_1_‐optimized geometries and the SMD solvation model. (n‐HXN: hexane, THF: tetrahydrofuran, DMF: N,N‐dimethylformamide, DMSO: dimethyl sulfoxide, EtOH: ethanol).

To further validate the SSC framework, the $\Delta {\hat{\nu }}_{\mathrm{emi}}^{*}$ values of various molecules in different solvents are compared with the corresponding experimental and DFT‐calculated values in Figure 3. The $\Delta {\hat{\nu }}_{\mathrm{emi}}^{*}$ values successfully reproduce the experimentally observed solvent‐dependent changes in emission energies, demonstrating that the SSC framework effectively captures solvatochromic shifts at the subgroup level. In particular, Figure 3B,E compare two naphthalene‐based molecules with subtle differences in substituents (cyanoethylene, tertiary amine, and isocyanide). The distinct $\Delta {\hat{\nu }}_{\mathrm{emi}}^{*}$ trends highlight the sensitivity of SSC to subtle structural variations. Similarly, Figure 3C,F show the solvent‐dependent emission energy shifts of two coumarin derivatives. As solvents change from non‐hydrogen‐bonding (NHB) to hydrogen‐bond donor (HBD) types, the $\Delta {\hat{\nu }}_{\mathrm{emi}}^{*}$ values not only reproduce experimental solvatochromic shifts but also assign chemically interpretable contributions to individual subgroups. Remarkably, even though the mean absolute error (MAE) of the SSC‐GAT model is ∼0.09 eV, the $\Delta {\hat{\nu }}_{\mathrm{emi}}^{*}$ values are able to resolve solvent‐induced changes in emission energies as small as 0.06 eV, highlighting the fine‐grained sensitivity of the SSC framework. In contrast, the DFT‐calculated values generally underestimate the magnitude of the solvatochromic shifts and fail to reproduce the solvatochromic shifts observed in different solvents, as shown in Figure 3.

Before closing this section, it is important to highlight the practical applicability of predicting the absorption and emission energies of organic molecules in solution using the learned NSC values. Because the current SSC‐based DL model was trained on experimental data for neutral organic molecules with molecular weights below 480 Da in solution, it provides reasonably accurate predictions for small neutral organic molecules in which all constituent subgroups collectively constitute a single chromophore or fluorophore unit. The applicability of the model is therefore primarily limited to this training domain. More complex molecular systems, including nanoparticles, molecular aggregates, charged compounds, organometallic compounds, and polymers, may fall outside its current applicability domain. Figure S11 presents representative predictions for donor–acceptor (D–A) molecules with molecular weights above 480 Da. The reduced predictive accuracy for these molecules can be attributed primarily to two factors: (1) the current subgroup definitions, based on the segmentation rule (6,2,0), may not adequately represent large molecular systems whose solvatochromic behavior cannot be captured by a single chromophore or fluorophore unit; and (2) molecules exhibiting negative solvatochromism are predicted less accurately because the training dataset is dominated by molecules showing positive solvatochromism [2]. A promising strategy for overcoming these limitations is to develop complementary, physics‐informed frameworks that extend the applicability domain of the SSC model while preserving its chemical interpretability.

### 2.9 Evaluation with the Kamlet‐Taft Method

To further validate the chemical interpretability of the SSC framework, we examined the correlation between normalized subgroup contribution derived from emission energies ($\delta {\hat{\nu }}_{\mathrm{emi}}^{*}$) and conventional solvent polarity parameters using the Kamlet‐Taft equation,

(5) $$\nu_{\max}=\nu_{0}+s\pi^{*}+a\alpha +b\beta$$

where *s*, *a*, and *b* are fitting coefficients representing the sensitivity of the molecular property to each solvent parameter, *ν*_0_ is the property value in a hypothetical reference solvent, and *ν*_max_ is the maximum transition (emission) energy (or wavenumber) of a molecule in a given solvent.

**FIGURE 4:**
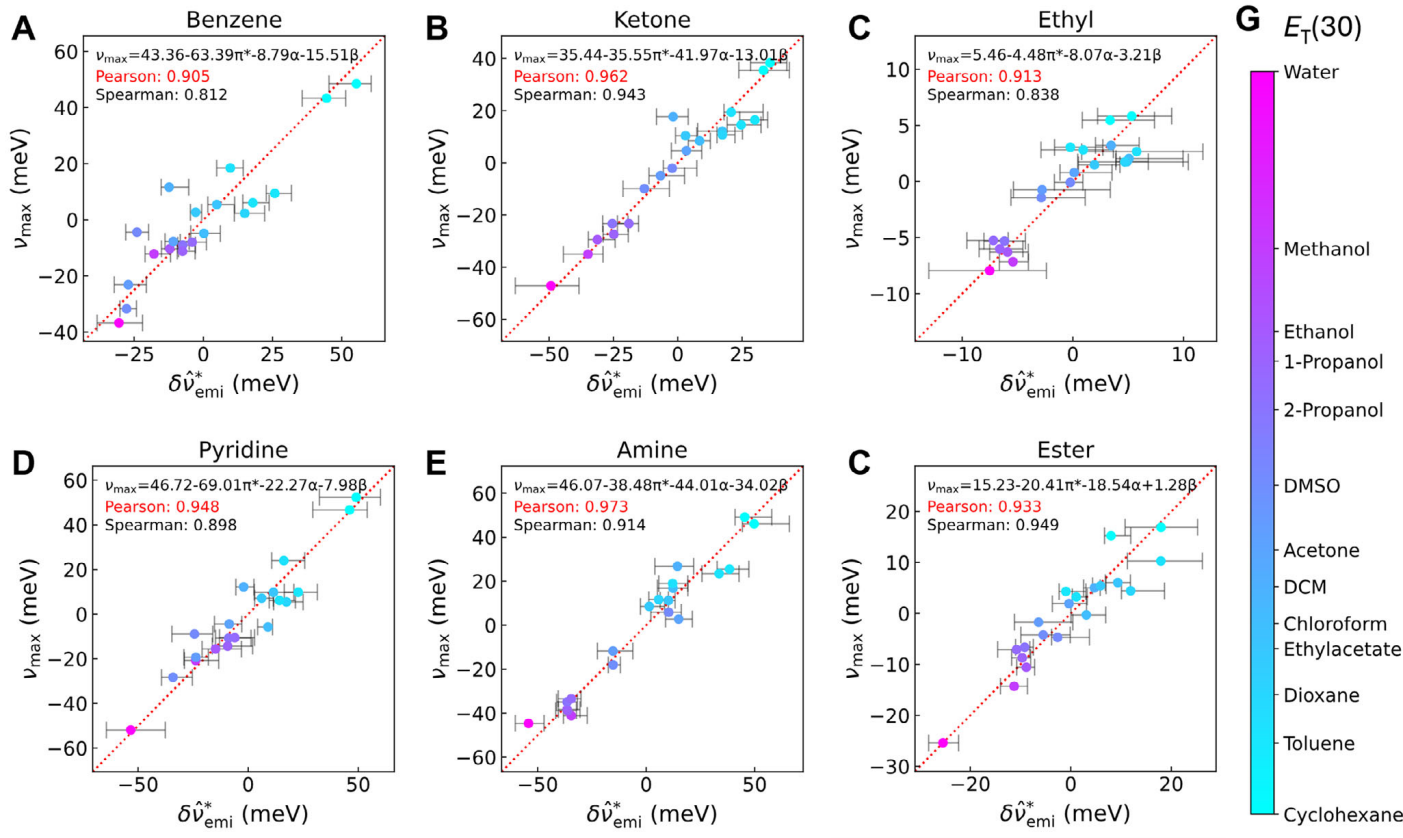
Linear regressions of NSC values to the Kamlet‐Taft equation (*ν*_max_ = *ν*_0_ + sπ* + *aα* + *bβ*) for representative subgroups: (A) benzene, (B) pyridine, (C) ethyl, (D) amine, (E) ketone, and (F) ester. Horizontal error bars denote the variance in $\delta {\hat{\nu }}^{*}$ values obtained from SSC‐GAT models initialized with different random seeds, reflecting the reproducibility of subgroup‐level contributions. (G) Solvent polarity parameters, represented by *E*_T_(30), are shown as a color scale for each data point.

If $\delta {\hat{\nu }}_{\mathrm{emi}}^{*}$ values faithfully capture molecule‐solvent interactions at the subgroup level, they should be describable by Equation (5). To test this hypothesis, we performed linear regressions of $\delta {\hat{\nu }}_{\mathrm{emi}}^{*}$ for representative subgroups against the Kamlet‐Taft parameters (Figure 4; additional examples in Figure S10). The successful fits, with high Pearson and Spearman correlation coefficients, demonstrate that $\delta {\hat{\nu }}_{\mathrm{emi}}^{*}$ values reproduce solvatochromic trends originally proposed by Kamlet and Taft, thereby validating the chemical consistency of the SSC framework.

Importantly, the fitted coefficients (*s*, *a*, and *b*) in the Kamlet and Taft equation provide mechanistic insights into subgroup‐solvent interactions. The relatively large 𝑠 values for benzene (–63.39) and pyridine (–69.01) indicate that their solvatochromic shifts are strongly governed by dipolarity‐polarizability (π*) interactions. For ketone and ester groups, the *a* coefficients (–41.97 and –18.54, respectively) are large, whereas the *b* coefficients (–13.01 and 1.28, respectively) are relatively small, consistent with their strong interactions with hydrogen‐bond donors but limited sensitivity to hydrogen‐bond acceptors. In contrast, amine groups show large values for both *a* (–44.01) and *b* (–34.02), reflecting their dual ability to participate in donor‐acceptor hydrogen‐bonding. By comparison, ethyl groups display weak dependence on all three Kamlet‐Taft solvent parameters (*s* = –4.48, *a* = –8.07, and *b* = –3.21), consistent with their chemically inert character toward polar interactions. A comprehensive set of fitted coefficients for additional subgroups is provided in Table S9.

### 2.10 Evaluation With the Bilot‐Kawski Method

**FIGURE 5:**
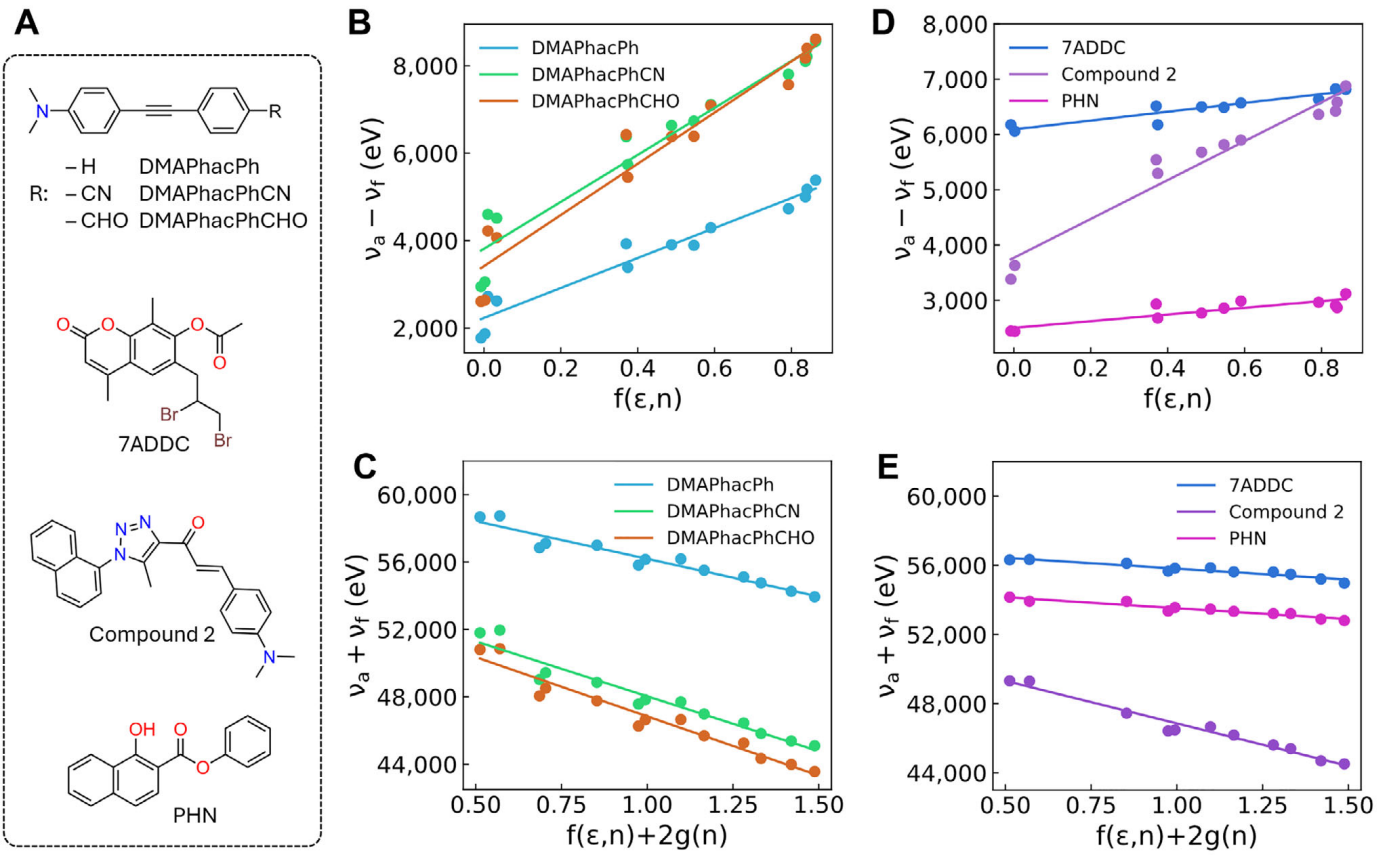
Evaluation of the SSC framework using the Bilot‐Kawski method. (A) Molecular structures of the benchmarking molecules studied. Plots of (ν_a_—ν_f_) against the solvent polarity function *f*(*ε*, *n*) for (B) DMAPhacPh derivatives [52], and for (D) 7ADDC [53], compound 2 [54], and PHN [55]. Plots of (ν_a_ + ν_f_) against *f*(*ε*, *n*) + 2*g*(*n*) for (C) DMAPhacPh derivatives, and for (E) 7ADDC, compound 2, and PHN.

Ground‐ and excited‐state dipole moments of molecules can be experimentally estimated using solvatochromic methods such as those proposed by Bilot‐Kawski [48], Lippert‐Mata [49, 50], and Bakhshiev [51]. Given the SSC framework's predictive capability for solvatochromic shifts, we evaluated its consistency with the Bilot‐Kawski method. Figure 5 demonstrates the potential of the SSC‐GAT model to estimate molecular dipole‐related properties based on predicted absorption and emission energies, while Table S6 summarizes the comparison of slopes obtained from SSC predictions with those reported from Bilot‐Kawski analyses. For most molecules, the predicted slopes show good agreement with reported values. While discrepancies are observed for molecules with steep experimental slopes, these likely arise from uncertainties in experimental fitting, which can be strongly influenced by the choice and number of solvent data points. The observed linear correlation between the SSC‐predicted values and the solvent polarity function confirms that the SSC model not only aligns with traditional solvatochromic methods but also captures the essential correlation between electronic transition energies and solvent polarity.

## 3 Conclusions

In this work, we developed deep learning models based on the solvatochromic subgroup contribution (SSC) framework, a chemistry‐informed deep learning approach for modeling the solvent‐dependent optical properties of molecules in solution. The SSC framework describes solvatochromic shifts through localized interactions between molecular subgroups and the surrounding solvent environment. Within this framework, solution‐phase electronic transition energies are decomposed into three chemically interpretable components: the reference property (RP), which represents a latent intrinsic molecular property; the subgroup contribution (SC), which captures direct molecule‐solvent interactions at the subgroup level; and the proximity effect factor (PEF), which accounts for the influence of neighboring subgroups on these interactions. This decomposition provides a physically motivated representation of solvent‐dependent electronic transition energies.

Trained on an experimental database of molecular absorption and emission energies measured in solution, the SSC‐based DL models achieved improved predictive accuracy and chemical interpretability compared with existing approaches, including concatenation‐based models, CIGIN, and MolMerger. More importantly, the learned SC values showed strong correlations with classical solvent‐polarity descriptors, including the *E*_T_(30) and Kamlet‐Taft parameters, and successfully reproduced Bilot‐Kawski relationships. These results demonstrate that the SSC framework captures physically meaningful aspects of molecule‐solvent interactions.

Overall, the SSC framework establishes a generalizable, interpretable, and data‐driven paradigm for incorporating solvent effects into AI‐driven molecular property prediction. By embedding chemical and physical insights into deep‐learning architectures, it enables explainable models that provide both accurate predictions and mechanistic insight into the solvatochromic effects governing molecular optical properties in solution. Although challenges remain in improving the reproducibility and chemical interpretability of the learned parameters and in expanding the applicability domain of the model, these limitations may be addressed through the continued development of physically informed modeling strategies. Beyond solvatochromism, the SSC framework may provide a transferable foundation for modeling other complex environment‐dependent phenomena, including solution‐phase reactions, photophysical processes, and catalysis.

## Author Contributions

J. Park developed the models, conducted the investigation and data analysis, and prepared the original draft. M. Han, K. Lee, and S. Kim contributed to data curation and validation. S. Park conceived the study, contributed to data analysis, reviewed and edited the manuscript, and supervised the project.

## Conflicts of Interest

The authors declare no conflicts of interest.

## Supporting information




**Supporting File 1**: advs77347‐sup‐0001‐SuppMat.docx.

**Supporting File 2**: advs77347‐sup‐0002‐SuppMat.csv.

**Supporting File 3**: advs77347‐sup‐0003‐SuppMat.csv.

## Data Availability

The data that support the findings of this study are available in the Supporting Information. Additionally, the datasets, Deep4Chem optical database [56], and the code used to train and evaluate the SSC‐based DL models are available at: https://github.com/ACCA‐KU/SSC.git. The repository includes all scripts required to reproduce the results of this study, including model training, molecular property prediction, and visualization of normalized subgroup contributions (NSC). The SSC‐GAT model has been implemented as a publicly accessible web application (http://ACCA.korea.ac.kr/explainable/SSC).
