# Supplementary material for: Interpretable Deep Learning of Molecule‐Solvent Interactions Using a Solvatochromic Subgroup Contribution Framework
Source: Adv Sci (Weinh). 2026 Aug 27;13(59):e77347. doi: 10.1002/advs.77347 (PMC13521183; doi:10.1002/advs.77347)
Supplement: Supplementary file 1 [file ADVS-13-e77347-s001.docx]

Supporting Information

**Interpretable Deep Learning of Molecule-Solvent Interactions Using a Solvatochromic Subgroup Contribution Framework**

Jinyong Park,^1^ Minhi Han,^1^ Kiwoong Lee,^1^ Seokwoo Kim,^1^ and Sungnam Park^1,2^*

^1^ Department of Chemistry and Center for Advanced Molecular Science, Korea University, Seoul, 02841, Korea

^2^ Deep4Chem Inc., Asan Science Building 223, 145 Anam-ro, Seongbuk-gu, Seoul, 02841, Korea

*E-mail: spark8@korea.ac.kr (S.P.)

**Deep learning models with the SSC method**

As illustrated in Figure 1, deep learning models with the SSC method predict the absorption and emission energies of molecules in a given solvent by decomposing the contributions into three terms: reference property (RP), subgroup contribution (SC), and proximity effect factor (PEF). The RP is related to the intrinsic absorption (or emission) energy of a molecule in the gas phase, independent of solvent effects, and is estimated solely from the molecular structure. Formally, it can be expressed as:

$$\tilde{v}_{\text{RP}}=f(\mathbf{GNN}(c))$$

where *c* is the molecular graph, **GNN**: *c* → ℝ*^n^* denotes any benchmark graph neural network (e.g., GCN, GAT, MPNN, AFP) that encodes the molecular graph into a latent feature, and *f:* ℝ*^n^* → ℝ is a feed-forward neural network composed of fully connected layers, activation functions, and dropout. The SC is defined for each combination of a molecular subgroup and a solvent:

$$\delta\tilde{v}_{i,\text{solv}}=f(\mathbf{GNN}(i)\oplus\mathbf{GNN}(s))$$

where *i* and *s* are the molecular graphs of a subgroup and solvent, respectively, and ⊕ denotes vector concatenation. Importantly, the SC for a given subgroup-solvent pair is consistent across different molecules (e.g., the phenyl group in toluene yields the same SC value regardless of the parent molecule). The PEF accounts for modifications of subgroup contributions due to the influence of neighboring subgroups (e.g., inductive effects, resonance delocalization, steric hindrance). The PEF is derived from a context vector that incorporates information from the molecular graph, the focal subgroup, and its adjacent subgroups in a given solvent:

$$P_{i}=f(j_{i}\oplus\mathbf{GNN}(s)\oplus\mathbf{GNN}(c))$$

where *j_i_* is the node feature of the junction tree corresponding to subgroup *i*. Finally, the predicted absorption (or emission) energy of a molecule in a given solvent is given by

$$\tilde{v}_{\text{solv}}=\tilde{v}_{\text{RP}}+\sum_{i} \left\{\delta\tilde{v}_{i,\text{solv}}\times P_{i}\right\}$$

as shown in Figure 1d. Notably, the SSC method is independent of the choice of GNN architecture, meaning that any conventional GNN can be seamlessly integrated within the framework.

**Detail Architecture of SSC-based model**

Figure S1 shows the deep learning model with the solvatochromic subgroup contribution (SSC) method proposed in this study. Taking the molecular graph *G*_S_, subgroup graphs *S*_S_, junction tree graph *J*_S_, atom feature matrix of molecule *A*_S_, bond feature matrix of molecule *B*_S_, solvent molecular graph *G*_L_, and atom feature matrix of solvent *A*_L_ as an input, SSC-based model firstly encodes the context vectors of a molecule *h*_S_, molecular subgroups *h*_sub_, and a solvent *h*_L_.

$$h_{\text{S}}=\text{R}_{\mathrm{GNN}}(\text{M}_{\mathrm{GNN}}(G_{\text{S}},A_{\text{S}},B_{\text{S}}))$$

$$h_{\text{sub},i}=\text{R}_{\mathrm{GNN}}(\text{M}_{\mathrm{GNN}}(S_{\text{S,}i},A_{\text{S,}i},B_{\text{S,}i}))$$

$$h_{\text{L}}=\text{R}_{\mathrm{G}\text{C}\mathrm{N}}(\text{M}_{\mathrm{G}\text{C}\mathrm{N}}(G_{\text{L}},A_{\text{L}}))$$

The subscript *i* indicates the corresponding variables for *i*^th^ molecular subgroup. The message passing functions M_GNN_ and reduction function R_GNN_ for encoding a molecule and its subgroups are varied depending on the selected graph neural networks (i.e., GCN, MPNN, GAT, DMPNN and AttentiveFP). Each of them is defined in following section. GCN was selected as the graph convolution algorithm for solvent molecules, regardless of the algorithms adopted for molecules, since the limited variety of solvents does not require sophisticated encoding techniques. To calculate PEF, graph convolution is conducted on the subgroup feature with junction tree graph, which represents the connectivity between subgroups.

$$H_{\text{cir}}=\text{M}_{\mathrm{G}\text{C}\mathrm{N}}(J_{\text{S}},H_{\text{sub}})$$

*i*^th^ row of matrix *H*_sub_ (or *H*_cir_) corresponds to *h*_sub_*_,i_* (or *h*_cir_*_,i_*). The SSC parameters, RF (*ṽ*_RP_), SC (δ*ṽ*), and PEF (*P*), are calculated using above encoded vectors.

$$\tilde{v}_{\text{RP}}=f(h_{\text{S}})$$

$\text{δ}\tilde{v}_{i,\text{solv}}=f([h_{\text{sub},i};h_{\text{L}}])$

$$P_{i}=f([h_{\text{cir},i};h_{\text{S}};h_{\text{L}}])$$

Function *f* represents a sequence of several linear layers, dropout layers, and activation functions (i.e. feed-forward neural network), and [;] indicates the concatenation of vectors. As a result, the final output of SSC-based model can be expressed as following expression.

$$\tilde{v}_{\text{solv}}=\tilde{v}_{\text{RP}}+\sum_{i} \left\{\text{δ}\tilde{v}_{i,\text{solv}}\times P_{i}\right\}$$

All graph convolutional networks are implemented using the Deep Graph Library (DGL) package.

It should be noted that different activation functions are used at the final layer of each feed-forward block of the deep learning model in Figure S1. The layer for RP omits an activation function for last step to allow unrestricted value prediction. For the SSC (subgroup contribution) layer, a hyperbolic tangent (tanh) activation is applied to constrain the outputs within the range of −1 to 1. Because the SC is empirically known to remain below 1 eV (see Extended Data Table 1), the tanh function prevents the divergence of SC values while promoting stable training. In contrast, the layer for the PEF adopts a sigmoid function followed by the multiplication by 2. As discussed in the main text, the PEF adjusts the SC to take into account the proximity effect. Because of this role, the sigmoid activation function ensures the PEF values to be between 0 and 2 as shown in Figure S9.

**Derivation of NSC values**

To enhance the reproducibility and robustness of SC values, we introduced the normalized solvatochromic contribution (NSC), which is defined as,

$\text{δ}\tilde{v}_{i,\text{solv}}^{*}=\text{δ}\tilde{v}_{i,\text{solv}}-\left\langle\text{δ}\tilde{v}_{i,\text{solv}}\right\rangle=\text{δ}\tilde{v}_{i,\text{solv}}-\frac{1}{n_{\{\text{solv}\}}}\sum_{s\in\{\text{solv}\}} \text{δ}\tilde{v}_{i,s}$ (S1)

where {solv} is the set of solvents used in this study (listed in below), and *n*_{solv}_ = 20. As shown in Figure S6, NSC values exhibit well-overlapping trends across independently trained models, demonstrating their robustness. Using NSC, we further define the normalized solvatochromic shift (Δ*ṽ**) of a molecule, representing the relative change in transition energy across solvents and expressed without the PEF term to enhance interpretability.

$\Delta\tilde{v}_{\text{solv}}^{*}=\sum_{i} \text{δ}\tilde{v}_{i,\text{solv}}^{*}$ (S2)

Comprehensive NSC values (δ*ṽ**) of all subgroups are provided in Supporting Data Set 1 (absorption energies) and 2 (emission energies). These values were derived from an ensemble of SSC-GAT models trained on the MW480 dataset with eight independent random seeds. To ensure reliability, two models whose SC values deviated significantly from the others were identified as outliers and excluded from further analysis. The SC values obtained from the models were normalized by subtracting the average of SC values in 20 solvents. The normalized values were then averaged to yield the final NSC values in each solvent. To assess robustness, the ensemble variance was also reported, providing a measure of consistency and reproducibility. This normalization enables extraction of chemically meaningful trends from SSC-derived contributions while reducing sensitivity to variability in RP predictions. In this study, NSC analysis focused on the 20 most frequently represented solvents in the dataset: dichloromethane (DCM), toluene, chloroform, tetrahydrofuran (THF), methanol, dimethyl sulfoxide (DMSO), ethanol, dimethylformamide (DMF), ethyl acetate (EA), hexane, cyclohexane (CH), water, dioxane, acetone, diethyl ether, benzene, 2-propanol, 1-butanol, and 1-propanol. These solvents span a broad range of polarity, hydrogen-bonding characteristics, and dielectric constants, enabling systematic evaluation of solvatochromic trends.

**Figure S1.** Schematic illustration of the deep learning model with the full SSC method. ⊕ represents the concatenation operation. In this study, the hidden feature dimension is set to 256.

**Implementation of benchmarking solvent-incorporation models**

In this section, the implementations of benchmarking solvent-incorporation models are briefly introduced from a mathematical perspective. This section provides how the benchmarking models work but may not provide exact definitions of them.

**Concatenation method:**

$$h_{\text{S}}=\text{R}_{\mathrm{GNN}}(\text{M}_{\mathrm{GNN}}(G_{\text{S}},A_{\text{S}},B_{\text{S}}))$$

$$h_{\text{L}}=\text{R}_{\mathrm{GNN}}(\text{M}_{\mathrm{GNN}}(G_{\text{L}},A_{\text{L}},B_{\text{L}}))$$

$$\hat{y}=f([h_{\text{S}};h_{\text{L}}])$$

**Figure S2.** (a) Concatenation method to model the solvent effect on the molecular properties. The molecular context vector and solvent context vector are linked together before the fully connected layer. (b) Schematic illustration of the deep learning model with the concatenation method. ⊕ represents the concatenation operation.

**Chemically interpretable graph interaction network (CIGIN)**^1^**:**

$$H_{\text{S}}=\text{M}_{\mathrm{GNN}}(G_{\text{S}},A_{\text{S}},B_{\text{S}})$$

$$H_{\text{L}}=\text{M}_{\mathrm{GNN}}(G_{\text{L}},A_{\text{L}},B_{\text{L}})$$

$$I=\mathrm{Tanh}(H_{\text{S}}\times H_{L}^{\text{T}})$$

Matrix *I* is an interaction map, which is expected to represent the specific interaction between atom-pair of the molecule and solvent.

$$\begin{matrix} H'_{\text{S}}=I\times H_{\text{L}} \\ H'_{\text{L}}=I^{\text{T}}\times H_{\text{S}} \end{matrix}$$

$$\begin{matrix} h_{\text{S}}=\text{R}(H_{\text{S}},H'_{\text{S}}) \\ h_{\text{L}}=\text{R}(H_{\text{S}},H'_{\text{S}}) \end{matrix}$$

Function *R* is a reduction function which can be Set2Set function or Sum-pooling function. In this study, Sum-pooling function is used.

$$\hat{y}=f([h_{\text{S}};h_{\text{L}}])$$

**MolMerger^2^:**

$$G_{\text{merged}}=\text{Merge}(G_{\text{S}},G_{\text{L}})$$

Function ‘Merge’ connects the atom with the most negative partial charge in the first molecule to the atom with the most positive partial charge in the second molecule, and vice versa. In this way, the two graphs are connected by two additional edges.

$$A_{\text{merged}}=[A_{\text{S}};A_{\text{L}}]$$

$$B_{\text{merged}}=[B_{\text{S}};B_{\text{new}};B_{\text{L}}]$$

Matrix *B*_new_ represents the features of additional edges, which are set to zero vectors.

$$h_{\text{merged}}=\text{R}_{\mathrm{GNN}}(\text{M}_{\mathrm{GNN}}(G_{\text{merged}},A_{\text{merged}},B_{\text{merged}}))$$

$$\hat{y}=f(h_{\text{merged}})$$

**Table S1**. Best model performances based on the solvent effects on absorption and emission energies of organic molecules.

| **Property** | **Solvation model** | **Model** | **MAE (eV)** | **RMSE (eV)** | **R2** |
| --- | --- | --- | --- | --- | --- |
| Absorption energy | SSC | GCN | 0.0887 | 0.1566 | 0.9456 |
|  |  | MPNN | 0.0905 | 0.1570 | 0.9425 |
|  |  | GAT | 0.0860 | 0.1506 | 0.9497 |
|  |  | DMPNN | **0.0844** | **0.1484** | **0.9512** |
|  |  | AttentiveFP | 0.0938 | 0.1621 | 0.9417 |
|  | Concat. | GCN | 0.1024 | 0.1644 | 0.9442 |
|  |  | MPNN | 0.0959 | 0.1620 | 0.9418 |
|  |  | GAT | 0.0905 | 0.1586 | 0.9442 |
|  |  | DMPNN | 0.0953 | 0.1712 | 0.9344 |
|  |  | AttentiveFP | 0.0956 | 0.1665 | 0.9385 |
|  | CIGIN | GCN | 0.0897 | 0.1550 | 0.9463 |
|  |  | MPNN | 0.1037 | 0.1625 | 0.9409 |
|  |  | GAT | 0.1001 | 0.1618 | 0.9398 |
|  |  | DMPNN | 0.0955 | 0.1623 | 0.9410 |
|  |  | AttentiveFP | 0.1049 | 0.1650 | 0.9391 |
|  | MolMerger | GCN | 0.0926 | 0.1615 | 0.9442 |
|  |  | MPNN | 0.0959 | 0.1562 | 0.9478 |
|  |  | GAT | 0.0954 | 0.1620 | 0.9438 |
|  |  | DMPNN | 0.0991 | 0.1703 | 0.9379 |
|  |  | AttentiveFP | 0.1005 | 0.1639 | 0.9425 |
| Emission energy | SSC | GCN | 0.0860 | 0.1348 | 0.9133 |
|  |  | MPNN | 0.0839 | 0.1363 | 0.9114 |
|  |  | GAT | **0.0805** | **0.1310** | **0.9181** |
|  |  | DMPNN | 0.0810 | 0.1339 | 0.9145 |
|  |  | AttentiveFP | 0.0899 | 0.1420 | 0.9039 |
|  | Concat. | GCN | 0.0941 | 0.1422 | 0.9035 |
|  |  | MPNN | 0.0967 | 0.1448 | 0.9001 |
|  |  | GAT | 0.0852 | 0.1359 | 0.9119 |
|  |  | DMPNN | 0.0868 | 0.1374 | 0.9078 |
|  |  | AttentiveFP | 0.0878 | 0.1414 | 0.9046 |
|  | CIGIN | GCN | 0.0859 | 0.1338 | 0.9125 |
|  |  | MPNN | 0.0866 | 0.1344 | 0.9118 |
|  |  | GAT | 0.0963 | 0.1492 | 0.8913 |
|  |  | DMPNN | 0.0833 | 0.1331 | 0.9139 |
|  |  | AttentiveFP | 0.0906 | 0.1387 | 0.9061 |
|  | MolMerger | GCN | 0.0841 | 0.1314 | 0.9176 |
|  |  | MPNN | 0.0881 | 0.1383 | 0.9088 |
|  |  | GAT | 0.0895 | 0.1376 | 0.9098 |
|  |  | DMPNN | 0.0917 | 0.1430 | 0.9024 |
|  |  | AttentiveFP | 0.0865 | 0.1367 | 0.9109 |

**Performance of SSC-based models across different dataset sizes**

The performance of deep learning models with the SSC method was evaluated using datasets with different molecular weight cutoffs: MW300, MW480, and All (see Methods and Figs. S3–S4 for dataset descriptions). Unless otherwise specified, the MW480 dataset was used for analysis in the main text, owing to its balance between dataset size and reproducibility (see discussion in the following sections). Table S2 summarizes the statistical performance of SSC-based models across the three datasets. In general, model accuracy decreased in the order MW480 > MW300 > All, reflecting the increasing structural and chemical diversity of molecules in larger datasets. Importantly, across all datasets, the SSC method consistently outperformed the concatenation method in modeling solvent effects.

**Table S2**. Mean absolute error (MAE) of each model evaluated on the test set

| **Property** | **Solvation model** | **DL model** | **MW300 (eV)** | **MW480 (eV)** | **All (eV)** |
| --- | --- | --- | --- | --- | --- |
| Absorption energy | Concat. | GCN | **0.0965 ± 0.0027** | 0.1046 ± 0.0023 | **0.1016** |
|  |  | MPNN | 0.1014 ± 0.0021 | 0.1026 ± 0.0067 | 0.1077 |
|  |  | GAT | 0.0980 ± 0.0028 | **0.0933 ± 0.0029** | 0.1102 |
|  |  | DMPNN | 0.1042 ± 0.0014 | 0.1028 ± 0.0028 | 0.1096 |
|  |  | AttentiveFP | 0.1031 ± 0.0008 | 0.0984 ± 0.0025 | 0.1147 |
|  | SSC | GCN | 0.1235 ± 0.0072 | 0.0911 ± 0.0018 | **0.1044** |
|  |  | MPNN | 0.1154 ± 0.0028 | 0.1012 ± 0.0050 | 0.1163 |
|  |  | GAT | **0.0996 ± 0.0016** | **0.0910 ± 0.0037** | 0.1056 |
|  |  | DMPNN | 0.1021 ± 0.0031 | 0.0943 ± 0.0028 | 0.1183 |
|  |  | AttentiveFP | 0.1052 ± 0.0036 | 0.1030 ± 0.0095 | 0.1104 |
| Emission energy | Concat. | GCN | 0.0972 ± 0.0050 | 0.1058 ± 0.0094 | 0.1048 |
|  |  | MPNN | 0.0963 ± 0.0070 | 0.1038 ± 0.0047 | 0.1065 |
|  |  | GAT | **0.0922 ± 0.0042** | 0.0921 ± 0.0040 | **0.0999** |
|  |  | DMPNN | 0.0978 ± 0.0041 | 0.0989 ± 0.0051 | 0.1136 |
|  |  | AttentiveFP | 0.0955 ± 0.0051 | **0.0905 ± 0.0016** | 0.1148 |
|  | SSC | GCN | 0.1017 ± 0.0041 | 0.0934 ± 0.0057 | **0.0944** |
|  |  | MPNN | 0.1022 ± 0.0018 | 0.1016 ± 0.0091 | 0.1076 |
|  |  | GAT | **0.0890 ± 0.0050** | **0.0892 ± 0.0064** | 0.0990 |
|  |  | DMPNN | 0.9100 ± 0.0021 | 0.0907 ± 0.0043 | 0.1038 |
|  |  | AttentiveFP | 0.1001 ± 0.0025 | 0.0960 ± 0.0085 | 0.0968 |

**Ablation study of the SSC method**

Table S3 summarizes the results of the ablation study on the SSC framework. To assess the role of the proximity effect factor (PEF), the SSC-GAT model was trained without PEF contributions (denoted SSC/PE), by fixing all PEF values to 1. This led to a noticeable decrease in predictive accuracy compared with the full SSC model, confirming that proximity effects from neighboring subgroups are non-negligible. Interestingly, when parameters pre-trained on the concatenation model were transferred into the SSC/PE model (denoted TL–SSC/PE), the prediction accuracy improved to levels comparable to the concatenation baseline. This suggests that even without PEF, the SSC formulation is at least as valid as concatenation for modeling solvent effects. The central goal of the SSC framework is to parameterize subgroup contributions to solvatochromism. However, because SSC simultaneously learns subgroup contributions (SC) and PEFs, the system becomes underdetermined: different SC and PEF combinations (e.g., doubling SC while halving PEF) can produce identical predictions. This ambiguity undermines the interpretability of SC values. To overcome this issue, we employed a two-step training strategy: first, the model was trained in SSC/PE mode to optimize SC values independently; second, the PEF values were optimized while freezing the RP and SC layers (denoted Frozen-SSC). The Frozen-SSC model achieves accuracy comparable to the full SSC model, while producing reproducible and chemically interpretable SC values. Although its accuracy is marginally lower than the full SSC model, a common trade-off between interpretability and predictive power, it remains superior to concatenation across most tasks. Therefore, all subsequent analyses in this study are based on Frozen-SSC, ensuring interpretability without sacrificing predictive performance.

**Table S3**. MAE of ablation study of the SSC-GAT model

| **Property** | **Solvation model** | **MW300 (eV)** | **MW480 (eV)** | **All (eV)** |
| --- | --- | --- | --- | --- |
| Absorption energy | Concat. | 0.0928 | 0.0905 | 0.1069 |
|  | SSC/PE | 0.1047 | 0.0958 | **0.1056** |
|  | TL-SSC/PE | 0.0987 | 0.0935 | 0.1120 |
|  | SSC | **0.0906** | **0.0860** | **0.1056** |
|  | Frozen-SSC | 0.0934 | 0.0898 | 0.1072 |
| Emission energy | Concat. | 0.0879 | 0.0852 | 0.0999 |
|  | SSC/PE | 0.0973 | 0.0971 | 0.1069 |
|  | TL-SSC/PE | 0.0932 | 0.0903 | 0.0955 |
|  | SSC | **0.0839** | **0.0805** | 0.0990 |
|  | Frozen-SSC | 0.0857 | 0.0848 | **0.0923** |

**Molecular input features**

To convert molecular structures from SMILES representations into molecular graph data, descriptors must be defined for both atoms (nodes) and bonds (edges). In this study, we employed six atomic features and two bond features, all obtained using the RDKit package. The selected features are listed in Table S4. Each feature was represented using one-hot encoding, ensuring compatibility with graph neural network architectures.

**Table S4.** Descriptor of atom for node features

| **Atom feature** | **Dimension** | **Description** |
| --- | --- | --- |
| Atomic number | 16 | The atomic number of the atom  C, N, O, S, H, F, Cl, Br, I, Se, Te, Si, P, B, Sn, Ge |
| Number of hydrogen atoms | 5 | Number of hydrogens of the atom defining from 0 to 4 |
| Number of covalent bonds | 7 | Number of covalent bonds defining from 0 to 6 |
| Aromaticity | 1 | Whether the atom has an aromaticity |
| Ring | 1 | Whether the atom belong to ring |
| Hybridization | 6 | Types of hybridization of the atom  s, sp, sp^2^, sp^3^, sp^3^d, sp^3^d^2^ |
| Formal charge | 9 | The electrical formal charge of atom defining from -4 to 4 |

**Table S5.** Descriptor of edge for edge features

| **Bond feature** | **Dimension** | **Description** |
| --- | --- | --- |
| Bond type | 4 | Types of bond:  single, double, triple, aromatic |
| *E-, Z*-Stereoisomer | 4 | STEREO_NONE, STEREO_ANY, STEREO_Z, STEREO_E |

**Experimental database**

To train the DL models based on the SSC framework, we used the Deep4Chem database of optical properties of organic compounds, which contains over 23,000 molecules in a variety of solvents (Figure S3). The optical data include electronic absorption and emission wavelengths, which were experimentally measured using UV-visible absorption and photoluminescence experiments, respectively. Specifically, absorption wavelengths correspond to the lowest-energy absorption peak (presumably, S_0_→S_1_ transition), and emission wavelengths correspond to the lowest-energy emission peak (S_1_→S_0_ transition) at room temperature. For consistency, all absorption and emission wavelengths (λ, nm) were converted into the corresponding transition energies (in eV) using the relation: *E*(eV) = 1239.8 / λ(nm). Because these transition energies of molecules in solution are strongly solvent-dependent, the dataset provides a rich source of information for modeling molecule-solvent interactions. The details of our experimental database used in this study are shown in Figure S3. To systematically evaluate model performance, we constructed three subsets of the database based on molecular weight: MW300 ( ≤ 300 g mol^-1^), MW480 (≤ 480 g mol^-1^), and All (no restriction). Unless otherwise stated, results presented in the main text correspond to the MW480 dataset, which balances dataset size with chemical diversity. Figure S4 shows a t-SNE distribution of molecular structures in the database using Morgan fingerprint descriptors, along with representative examples.

**Figure S3.** Statistics of the experimental database of absorption and emission energies of organic molecules in solution.

**Figure S4.** The *t*-SNE plot of molecular structures in our experimental database using Morgan fingerprints

**Model training and optimization**

We evaluated five message-passing deep learning (DL) architectures for graph neural networks (GNNs): graph convolution network (GCN),^3^ graph attention network (GAT),^4^ message-passing neural network (MPNN),^5^ directed-message passing neural network (DMPNN),^6^ and attentive fingerprint network (AttentiveFP).^7^ Based on empirical observations that model generalizability is critical for optical property datasets, we focused on these relatively simple and well-established architectures rather than recently proposed, more complex models. All models were implemented using PyTorch^8^ and the Deep Graph Library (DGL).^9^ Training employed the Adam optimizer with an initial learning rate of 0.001. To mitigate overfitting, we applied a learning rate scheduler and early stopping based on validation loss. The loss function was defined as the mean squared error (MSE) between predicted and experimental values. Atomic and bond descriptors used as input features are summarized in Tables S4 and S5.

The dataset of molecule/solvent pairs was randomly split into training, validation, and test sets in an 8:1:1 ratio. Target values were standardized by subtracting the mean and dividing by the variance of the dataset. Hyperparameters, including the number of GNN layers (3, 4, 5, or 6), hidden feature dimensions (64, 128, or 256), and dropout rates (0, 0.1, or 0.2), were optimized for each model using grid search. To assess reproducibility, the optimized models were retrained multiple times: eight times with the MW300 dataset, four times with the MW480 dataset, and once with the All dataset. This repeated training ensured that reported model performance was not the result of random initialization but reflected consistent predictive behavior across independent runs.

**Reproducibility of RP**

Figure S5 shows examples of the distribution of RP values obtained across independently trained models. In general, larger datasets tend to yield smaller RP values with reduced variance, suggesting that RP might converge as dataset size increases. However, this trend is not universal, and notable inconsistencies remain across different molecules. Even in the All dataset, the variance of RP values is significantly larger than the model’s MAE, indicating that RP does not converge in a reproducible manner. This lack of reproducibility does not undermine the validity of the SSC framework. Instead, it reflects the inherent challenge of estimating a gas-phase reference property that is experimentally inaccessible and mathematically underdetermined in a standard deep learning framework. While RP values may remain elusive in terms of strict reproducibility, they may still encode chemically meaningful latent information that contributes to accurate modeling of solvent-dependent properties.

**Figure S5**. Reproducibility of RP for emission energies of organic molecules.

**Reproducibility of SC values**

Figure S6 and S7 show the variations in SC and NSC values for several subgroups across different datasets. As shown on the left side of Figure S6 and S7, the absolute values of SC are not reproducible across the different initialization seeds. However, the right side of Figure S6 shows reasonably reproducible NSC values with different initialization seeds and different training datasets. In contrast, Figure S7 demonstrates that NSC values exhibit high variance across the different initialization seeds, and the trends differ significantly depending on the dataset.

**Figure S6**. Reproducibility of SC (left) and NSC (right) values for representative subgroups.

**Figure S7**. Reproducibility of SC (left) and NSC (right) values for representative subgroups.

**Reproducibility of PEF**

One remaining limitation is the slight variation in the scale of normalized subgroup contribution (NSC) depending on the training set. We attribute this variation to differences in frontier molecular orbital (FMO) localization associated with molecular size.^10,11^ As shown in Figure S8, the calculated FMOs for representative small and large molecules illustrate this trend. For the small molecule (Figure S8a), the FMOs are delocalized across nearly the entire molecular framework, suggesting that all constituent subgroups contribute to the overall optical response. Consequently, the solvent effect on each subgroup is reflected more uniformly in the molecular properties. In contrast, the FMOs of the large molecule (Figure S8b) are more localized, indicating that not all subgroups contribute equally to the molecular response. This interpretation is supported by the PEF distributions in Figure S9b, where large molecules generally exhibit smaller PEFs than their smaller counterparts. Furthermore, comparing Figure S9a and S9b reveals that the PEF derived from the All dataset is smaller for large molecules than that from the MW300 dataset. This suggests that models trained on larger molecules successfully capture the fact that subgroup contributions are relatively minor in extended systems, whereas models trained only on smaller molecules tend to assign consistent PEF values regardless of molecular size, as they lack exposure to this distinction. At this stage, in the absence of a definitive chemical reference for validating which representation is more appropriate, we adopt the NSC derived from the MW480 dataset for all subsequent analyses presented in the main text. Importantly, this choice ensures consistency in the comparative framework while preserving the robustness of PEF as a reliable descriptor across molecular scales

**Figure S8.** Frontier molecular orbitals of (a) a small molecule and (b) a large molecule obtained by DFT calculations (B3LYP/6-31G(d)). The figure illustrates the general trend of increased orbital localization often observed in larger molecules.

**Figure S9.** Distribution of PEF values grouped by molecular weight, derived from models trained on (a) the MW300 dataset and (b) the All dataset. Large molecules generally exhibit smaller PEFs in panel (b), indicating that models trained on larger systems capture reduced subgroup contributions compared to those trained on smaller systems.

**Kamlet-Taft method**

In this method, three parameters are used to characterize solvent effects: *α* (hydrogen-bond donating ability), *β* (hydrogen-bond accepting ability), and *π** (dipolarity-polarizability).^12-15^ The general Kamlet-Taft formalism for solvatochromism is expressed as

$$\nu_{\text{max}}=\nu_{0}+s\pi*+a\alpha+b\beta$$

where *s*, *a*, and *b* are fitting coefficients representing the sensitivity of the molecular property to each solvent parameter, *v*_0_ is the property value in a hypothetical reference solvent, and *ν*_max_ is the maximum transition (emission) energy (or wavenumber) of a molecule in a given solvent. Using *ν*_max_ values in different solvents and the Kamlet-Taft solvent parameters *α*, *β*, and *π**, the fitting coefficients *v*_0_, *s*, *a*, and *b* can be readily obtained.

For extracting the fitting coefficients, linear regressions of normalized solvatochromic shifts (δ*ṽ**) based on the Kamlet-Taft equation were performed using the LinearRegression function from the *scikit-learn* library.^16^ The Kamlet-Taft solvent parameters, *α* (hydrogen-bond donating ability), *β* (hydrogen-bond accepting ability), and *π** (dipolarity–polarizability), were obtained from their original literature sources (Table S7). For solvents with missing parameter values, the corresponding terms were set to zero to ensure consistency across the dataset. The error bars shown in Figure 4 represent the range (minimum to maximum) of NSC values derived from six independently trained SSC-GAT models with different random initializations, thereby reflecting the reproducibility of subgroup-level contributions.

**Figure S10**. Additional examples of linear regression using the Kamlet-Taft equation (*ν*_max_ = *ν*_0_ + *sπ** + *aα* + *bβ*).

**Bilot-Kawski method**

According to the Bilot-Kawski equation,^17^ the difference or sum of the absorption and emission wavenumbers is proportional to a solvent polarity function. These relationships are expressed as:

$$\tilde{\nu}_{\text{a}}-\tilde{\nu}_{\text{f}}=m^{(1)}f(\varepsilon,n)+\text{constant}$$

$$\tilde{\nu}_{\text{a}}+\tilde{\nu}_{\text{f}}=-m^{(2)}[f(\varepsilon,n)+2g(n)]+\text{constant}$$

$m^{(1)}=\frac{2(\mu_{\text{e}}-\mu_{\text{g}}\text{)}^{2}}{hca^{3}}$, $m^{(2)}=\frac{2(\mu_{\text{e}}^{2}-\mu_{\text{g}}^{2})}{hca^{3}}$

$f(\varepsilon,n)=\frac{2n^{2}+1}{n^{2}+2}\left(\frac{\varepsilon-1}{\varepsilon+2}-\frac{n^{2}-1}{n^{2}+2}\right)$, $g(n)=\frac{3}{2}\left(\frac{n^{4}-1}{(n^{2}+2)^{2}}\right)$

where *a* is the Onsager interaction radius of the molecule, *n* is the refractive index, and *ε* is the dielectric constant of the solvent, and *μ*_g_ and *μ*_e_ represent the ground- and excited-state dipole moments of the molecule, respectively. The solvent-specific values used in these calculations are listed in Table S7.

Linear regressions were performed using the LinearRegression function from the *scikit-learn* library. For the Bilot–Kawski analysis, a subset of solvents was employed, excluding hydrogen-bond donor (HBD) solvents, specifically water, methanol, ethanol, 1-propanol, 2-propanol, and 1-butanol, as well as tetrahydrofuran (THF). This exclusion follows prior studies reporting nonlinear behavior in these solvents, which makes them unsuitable for linear regression under the Bilot-Kawski approach.^18-21^

**Table S6**. Statistical parameters obtained from the Bilot-Kawski equation

| **Compound** | **Reported m^(1)^**  **(cm^-1^)** | **Predicted m^(1)^**  **(cm^-1^)** | **R^2^** | **Reported m^(2)^**  **(cm^-1^)** | **Predicted m^(2)^**  **(cm^-1^)** | **R^2^** | **Ref** |
| --- | --- | --- | --- | --- | --- | --- | --- |
| DMAPhacPh^18^ | 3659 | 3428 | 0.9457 | -6791 | -4474 | 0.9368 | [18] |
| DMAPhacPhCN^18^ | 8199 | 5368 | 0.9395 | -8403 | -6523 | 0.9361 | [18] |
| DMAPhacPhCHO^18^ | 9001 | 5853 | 0.9396 | -8183 | -7030 | 0.9466 | [18] |
| 7ADDC^19^ | 828 | 800 | 0.8783 | -1038 | -1257 | 0.9035 | [19] |
| Compound 2^20^ | 2344 | 3517 | 0.9517 | 3497 | -4923 | 0.9703 | [20] |
| PHN^21^ | 1137 | 605 | 0.7663 | -1204 | -1271 | 0.9216 | [21] |

**Table S7.** Dielectric constant, Reichardt’s solvent polarity parameters, *E*_T_(30), and Kamlet-Taft solvent parameters, *π**, *α*, and *β*.

| **Solvent** | **Dielectric constant (*ε*)** | ***E*_T_(30)**^22^ | *ν*_max_ = *ν*_0_ + *sπ** + *aα* + *bβ* | | |
| --- | --- | --- | --- | --- | --- |
|  |  |  | *π** ^13^ | *α* ^12^ | *β* ^14^ |
| dichloromethane | 8.93 | 40.7 | 0.5 |  |  |
| toluene | 2.4 | 33.9 | 0.535 |  |  |
| chloroform | 4.8 | 39.1 | 0.76 |  |  |
| acetonitrile | 37.5 | 45.6 | 0.713 | 0.29 |  |
| tetrahydrofuran | 7.5 | 37.5 | 0.513 |  | 0.523 |
| methanol | 33 | 55.4 | 0.586 | 0.99 | 0.62 |
| dimethylsulfoxide | 46.7 | 45.1 | 1 |  | 0.752 |
| ethanol | 24 | 51.8 | 0.54 | 0.85 | 0.77 |
| dimethylformamide | 37 | 43.2 | 0.875 |  | 0.71 |
| ethylacetate | 6 | 38 | 0.48 |  | 0.481 |
| hexane | 1.9 | 30.9 | -0.081 |  |  |
| cyclohexane | 2 | 30.8 | 0 |  |  |
| water | 80.1 | 63.1 | 1.09 | 1 | 0.14 |
| 1,4-dioxane | 2.25 | 36 | 0.553 |  | 0.386 |
| acetone | 21 | 42.3 | 0.683 |  | 0.499 |
| diethyl ether | 4.3 | 34.5 | 0.273 |  | 0.488 |
| benzene | 2.3 | 34.3 | 0.588 |  |  |
| 2-propanol | 19 | 48.4 | 0.505 | 0.687 | 0.92 |
| 1-butanol | 17.5 | 49.7 | 0.503 | 0.71 | 0.85 |
| 1-propanol | 22 | 50.5 | 0.534 | 0.766 | 0.9 |

**Table S8.** Solvent descriptors and solvent polarity function used in the Bilot-Kawski equation^17^

| **Solvent** | **refractive index (*n*)** | **Dielectric constant (*ε*)** | **f** | **g** | **φ** |
| --- | --- | --- | --- | --- | --- |
| dichloromethane | 1.424 | 9.02 | 0.590 | 0.287 | 1.165 |
| toluene | 1.497 | 2.43 | 0.033 | 0.335 | 0.703 |
| chloroform | 1.446 | 4.89 | 0.370 | 0.302 | 0.974 |
| acetonitrile | 1.344 | 36 | 0.865 | 0.234 | 1.331 |
| dimethylsulfoxide | 1.479 | 46.71 | 0.840 | 0.323 | 1.488 |
| dimethylformamide | 1.43 | 37.06 | 0.836 | 0.291 | 1.419 |
| ethylacetate | 1.372 | 6.03 | 0.488 | 0.253 | 0.994 |
| hexane | 1.375 | 1.89 | 0.002 | 0.255 | 0.512 |
| cyclohexane | 1.426 | 2.02 | -0.001 | 0.28 | 0.570 |
| 1,4-dioxane | 1.422 | 2.27 | 0.050 | 0.286 | 0.622 |
| acetone | 1.359 | 21.36 | 0.792 | 0.244 | 1.281 |
| diethyl ether | 1.352 | 4.42 | 0.374 | 0.239 | 0.853 |
| benzene | 1.501 | 2.4 | 0.009 | 0.338 | 0.686 |

**Table S9.** Coefficients of the Kamlet-Teft equation, *ν*_max_ = *ν*_0_ + *sπ** + *aα* + *bβ*, for frequently encountered subgroups, along with the correlation coefficients (R^2^) between the NSC values ($\Delta\hat{v}_{\text{emi}}^{*}$, meV) and *ν*_max_ (meV).

| **Subgroup** | **SMILES** | ***s*** | ***a*** | ***b*** | **R^2^** |
| --- | --- | --- | --- | --- | --- |
| Benzene | c1ccccc1 | -63.39 | -8.79 | -15.51 | 0.9048 |
| Methyl | *C | 0.46 | -3.92 | -1.32 | 0.6165 |
| Ketone | *=O | -35.55 | -41.97 | -13.01 | 0.9621 |
| Fluorine | *F | 10.14 | 14.63 | -1.54 | 0.8850 |
| Methoxy | *OC | 7.75 | -9.53 | 17.26 | 0.5144 |
| Alcohol | *O | 19.07 | 3.25 | 26.85 | 0.7736 |
| Dimethylamine | *N(C)C | -140.49 | -26.33 | -57.44 | 0.9035 |
| Ester | *OC(*)=O | -20.41 | -18.54 | 1.29 | 0.9339 |
| Cyano | *C#N | -43.80 | 1.40 | -6.33 | 0.8467 |
| Ethyl | *CC | -4.48 | -8.07 | -3.21 | 0.9127 |
| Chlorine | *Cl | -3.42 | -2.027 | 0.81 | 0.4074 |
| Alkene | *C=C* | -57.34 | 3.95 | -6.20 | 0.8797 |
| Thiophene | c1ccsc1 | -31.76 | -10.29 | 2.05 | 0.9331 |
| Chromene | c1ccc2occcc2c1 | -37.00 | -11.24 | -9.25 | 0.9407 |
| Primary amine | *N | -38.48 | -44.00 | -34.02 | 0.9733 |
| Diethyl amine | CCNCC | -83.82 | -22.59 | -49.12 | 0.9446 |
| Naphthalene | c1ccc2ccccc2c1 | -79.69 | -29.00 | -12.76 | 0.9765 |
| Trifluoromethyl | *C(F)(F)F | -76.07 | -9.94 | -57.94 | 0.9408 |
| Bromine | *Br | -7.66 | -7.05 | -0.06 | 0.8479 |
| Carboxylic acid | *C(=O)O | -13.69 | 4.95 | -7.50 | 0.4244 |
| Nitro | *[N+](=O)[O-] | -78.81 | -35.13 | -27.43 | 0.9042 |
| Tertiary amine | *N(*)* | -27.67 | -14.98 | -16.43 | 0.8993 |
| Pyridine | c1ccncc1 | -69.01 | -22.27 | -7.98 | 0.9481 |
| Butyl | *CCCC | -45.95 | -16.03 | 14.31 | 0.8534 |
| Tert-butyl | *C(C)(C)C | -54.43 | -45.44 | -16.29 | 0.9784 |
| Alkyne | *C#C* | -158.35 | -5.69 | -37.34 | 0.8979 |
| Aldehyde | *C=O | -92.17 | -79.13 | -37.62 | 0.9260 |
| 1,1-dicyanoethylene | C=C(C#N)C#N | -39.51 | -1.41 | -16.52 | 0.9001 |
| Alkoxide | *[O-] | -49.66 | -23.48 | -16.07 | 0.9001 |
| Triazole | c1cnnn1 | -77.30 | -47.72 | -9.19 | 0.9745 |
| Secondary amine | *N* | -31.37 | -8.21 | -15.26 | 0.9344 |

**Figure S11.** Predicted normalized solvatochromic shifts ($\Delta\hat{v}_{\text{emi}}^{*}$) for the molecules outside training domain compared with experimental values.^23-25^

**Segmentation of a molecule into its substructures (subgroups)**

To decompose molecules into substructures (subgroups), we employed the segmentation rule described in detail in our previous work.^26,27^ This algorithm provides a flexible framework for defining molecular substructures through three integer parameters, (*s*, *c*, *a*), which determine the segmentation hierarchy. By varying these parameters, molecules can be decomposed at different levels of structural resolution, including individual atoms, functional groups, and cyclic or conjugated units. This flexibility enables the subgroup definitions to be tailored to the molecular property of interest by controlling the separation, combination, or integration of specific structural motifs. A complete description of the algorithm, together with representative examples, is provided in our previous work.^26^

In this study, we adopted the segmentation rule (6,2,0), which preserves aromatic ring systems and generates molecular substructures broadly consistent with conventional functional groups, as illustrated in Figure S12. Under this scheme, fused and extended aromatic systems are treated as single subgroups. This definition reflects the assumption that electronic transition energies, including absorption and emission energies, are governed by the overall conjugated unit rather than by individual aromatic rings considered separately. The segmentation rule (6,2,0) therefore provides chemically meaningful subgroups for predicting absorption and emission energies within the SSC framework.

**Table S10**. **Predictive performance of models constructed using different segmentation rules**

| **Segmentation rule** | **MAE** | **RMSE** | **R^2^** |
| --- | --- | --- | --- |
| (4,0,1) | 0.0822 ± 0.0024 | **0.1322** ± 0.0025 | 0.9165 ± 0.0031 |
| **(6,2,0)**^†^ | **0.0810** ± 0.0009 | 0.1332 ± 0.0009 | **0.9165** ± 0.0012 |
| (9,4,4) | 0.0828 ± 0.0010 | 0.1355 ± 0.0008 | 0.9124 ± 0.0011 |
| (9,5,6) | 0.0857 ± 0.0004 | 0.1364 ± 0.0009 | 0.9092 ± 0.0012 |

Each metric was obtained from four independent training runs.

^†^The adopted segmentation rule in this work

**Figure S12.** Representative examples of molecular segmentation using different segmentation rules. The segmentation rule (6,2,0) preserves aromatic ring systems and generates molecular substructures that broadly correspond to conventional organic functional groups, thereby providing chemically meaningful subgroups for predicting absorption and emission energies within the SSC framework.

**Fig S13**. Parity plots, learning curves, and hyperparameters of SSC- and concatenation-based models trained on the emission energy (MW480) dataset, showing model performance and training behavior.

**Fig S14**. Parity plots, learning curves, and hyperparameters of CIGIN- and MolMerger-based models trained on the emission energy (MW480) dataset, showing model accuracy and training performance.

**Fig S15**. Parity plots, learning curves, and hyperparameters of SSC- and concatenation-based models trained on the absorption energy (MW480) dataset, showing model performance and training behavior.

**Fig S16**. Parity plots, learning curves, and associated hyperparameters of CIGIN- and MolMerger-based models trained on the absorption energy (MW480) dataset, showing model accuracy and training performance.

**References**

1	Pathak, Y., Laghuvarapu, S., Mehta, S. & Priyakumar, U. D. in *Proceedings of the AAAI Conference on Artificial Intelligence.* 873-880.

2	Ramani, V. & Karmakar, T. Graph Neural Networks for Predicting Solubility in Diverse Solvents Using MolMerger Incorporating Solute–Solvent Interactions. *J. Chem. Theory Comput.* **20**, 6549-6558 (2024).

3	Kipf, T. N. & Welling, M. Semi-supervised classification with graph convolutional networks. *arXiv preprint arXiv:1609.02907* (2016).

4	Velickovic, P. *et al.* Graph attention networks. *stat* **1050**, 10-48550 (2017).

5	Gilmer, J., Schoenholz, S. S., Riley, P. F., Vinyals, O. & Dahl, G. E. in *International conference on machine learning.* 1263-1272 (PMLR).

6	Yang, K. *et al.* Analyzing learned molecular representations for property prediction. *J. Chem. Inf. Model.* **59**, 3370-3388 (2019).

7	Xiong, Z. *et al.* Pushing the boundaries of molecular representation for drug discovery with the graph attention mechanism. *J. Med. Chem.* **63**, 8749-8760 (2019).

8	Pytorch. [*https://github.com/pytorch/pytorch*](https://github.com/pytorch/pytorch), 2017).

9	Minjie Wang, D. Z., Zihao Ye, Quan Gan, Mufei Li, Xiang Song, Jinjing Zhou, Chao Ma, Lingfan Yu, Yu Gai, Tianjun Xiao, Tong He, George Karypis, Jinyang Li, Zheng Zhang. Deep Graph Library: A Graph-Centric, Highly-Performant Package for Graph Neural Networks. *arXiv.org* (2019).

10	Xu, Y. *et al.* Frontier molecular orbital engineering: Constructing highly efficient narrowband organic electroluminescent materials. *Angew. Chem.* **135**, e202312451 (2023).

11	Chen, X.-K. A quantum-chemical insight into the role of charge-transfer states in organic emitters for electroluminescence. *CCS Chem.* **2**, 1256-1267 (2020).

12	Taft, R. & Kamlet, M. J. The solvatochromic comparison method. 2. The α-scale of solvent hydrogen-bond donor (HBD) acidities. *J. Am. Chem. Soc.* **98**, 2886-2894 (1976).

13	Kamlet, M. J., Abboud, J. L. & Taft, R. The solvatochromic comparison method. 6. The π* scale of solvent polarities. *J. Am. Chem. Soc.* **99**, 6027-6038 (1977).

14	Kamlet, M. J. & Taft, R. The solvatochromic comparison method. I. The β-scale of solvent hydrogen-bond acceptor (HBA) basicities. *J. Am. Chem. Soc.* **98**, 377-383 (1976).

15	Waghorne, W. E. A study of Kamlet–Taft β and π* scales of solvent basicity and polarity/polarizability using computationally derived molecular properties. *J. Solution Chem.* **49**, 466-485 (2020).

16	Pedregosa F, V., Ga"el, Gramfort A, Michel V, Thirion B, Grisel O, et al. Scikit-learn: Machine learning in Python. *J. Mach. Learn. Res.* **2825–2830** (2011).

17	Kawski, A. On the estimation of excited-state dipole moments from solvatochromic shifts of absorption and fluorescence spectra. *Z. Naturforsch. A* **57**, 255-262 (2002).

18	Szyszkowska, M., Bylińska, I. & Wiczk, W. Influence of an electron-acceptor substituent type on the photophysical properties of unsymmetrically substituted diphenylacetylene. *J. Photoch. Photobio. A* **326**, 76-88 (2016).

19	Sıdır, Y. G. & Sıdır, I. Solvent effect on the absorption and fluorescence spectra of 7-acetoxy-6-(2, 3-dibromopropyl)-4, 8-dimethylcoumarin: determination of ground and excited state dipole moments. *Spectrochim. Acta A* **102**, 286-296 (2013).

20	Singh, H., Sindhu, J. & Khurana, J. M. Determination of dipole moment, solvatochromic studies and application as turn off fluorescence chemosensor of new 3-(4-(dimethylamino) phenyl)-1-(5-methyl-1-(naphthalen-1-yl)-1H-1, 2, 3-triazol-4-yl) prop-2-en-1-one. *Sens. Actuators B Chem.* **192**, 536-542 (2014).

21	Sıdır, İ. & Sıdır, Y. G. Solvatochromism and intramolecular hydrogen-bonding assisted dipole moment of phenyl 1-hydroxy-2-naphthoate in the ground and excited states. *J. Mol. Liq.* **221**, 972-985 (2016).

22	Cerón‐Carrasco, J. P. *et al.* Solvent polarity scales: determination of new ET (30) values for 84 organic solvents. *J. Phys. Org. Chem.* **27**, 512-518 (2014).

23	Ortega, E. *et al.* Synthesis, characterization and photo physical-theoretical analysis of D-π-A compounds. 2. Chain length effect through even-odd effect on the photophysical properties. *Dyes Pigments* **147**, 75-82 (2017).

24	Thanikachalam, V., Jeeva, P. & Jayabharathi, J. Hybridised‐Local and Charge‐Transfer Excited States in Donor‐Spacer‐Acceptor Molecules for Efficient OLEDs: Combined Experimental and Theoretical Study. *ChemistrySelect* **2**, 1860-1870 (2017).

25	Chen, G. *et al.* A multifunctional emitter with synergistical adjustment of rigidity and flexibility for high-performance data-recording and organic light-emitting devices with “hot exciton” channel. *Chem. Eng. J.* **479**, 147616 (2024).

26	Park, J., Han, M., Lee, K. & Park, S. Hierarchical Graph Attention Network with Positive and Negative Attentions for Improved Interpretability: ISA-PN. *J. Chem. Inf. Comput.* (2024).

27	Han, M., Yokoo, T., Park, J., Oyaizu, K. & Park, S. Deep learning prediction of ionic conductivity in polymer electrolytes using hierarchical polymer graphs. *Chem. Eng. J.*, 166829 (2025).
